# Functional, structural, and taxonomical diversity of glycoside transporters in bacteria

**DOI:** 10.1093/femsre/fuag040

**Published:** 2026-09-02

**Authors:** Zhi Wang, Alexandra S Tauzin, Nicolas Terrapon, Matthieu Arlat, Xiaoqian Li, Gabrielle Potocki-Veronese, Aurore Labourel

**Affiliations:** TBI, Université de Toulouse, CNRS, INRAE, INSA, Toulouse, France; TBI, Université de Toulouse, CNRS, INRAE, INSA, Toulouse, France; Architecture et Fonction des Macromolécules Biologiques, CNRS, INRAE and Aix-Marseille University, Marseille, France; LIPME, INRAE/CNRS UMR0441/2594, Université de Toulouse, Université Paul Sabatier Toulouse 3, Castanet-Tolosan 31320, France; TBI, Université de Toulouse, CNRS, INRAE, INSA, Toulouse, France; TBI, Université de Toulouse, CNRS, INRAE, INSA, Toulouse, France; TBI, Université de Toulouse, CNRS, INRAE, INSA, Toulouse, France

**Keywords:** glycoside transporters, oligosaccharides, TonB-dependent transporters, ATP-binding cassettes (ABC), phosphotransferase system (PTS), major facilitator superfamily (MFS)

## Abstract

Glycoside metabolization is a crucial function in bacteria and a key feature in fermentation and synthetic biology. To feed on various glycosides with complex structures, bacteria have developed very diverse mechanisms to recognize, transport, and degrade them. Despite their crucial role, transporters are hard to identify and study due to challenges in expressing membrane proteins in recombinant form and the fact that most bacteria are uncultivable. In this review, we present the available methods and technologies for identifying and characterizing bacterial glycoside transporters. We consolidate existing knowledge on experimentally validated glycoside transporters from different families, including TonB-dependent transporters, ATP-binding cassette transporters, the phosphotransferase system, and the major facilitator superfamily, highlighting their taxonomic, functional, mechanistic, and structural diversity. The disparity between the number of functionally characterized glycoside transporters and the vast amount of sequence data available underlines the need for more efficient approaches to determine their specificity and to better understand the molecular mechanisms of glycoside utilization in bacteria.

## Teaser

The article highlights new methods to identify and study bacterial transporters involved in the uptake of glycosides, addressing challenges in understanding their diversity and function.

## Introduction

Carbohydrates are one of the major biological macromolecules found in living systems. They seldom occur in nature as monosaccharides (Prestegard et al. 2017). Instead, glycosyl units are mostly linked to each other, or to other non-glycosyl groups, to form glycosides (Cummings and Stephen 2007). Glycosides are structurally highly diverse because of the variety of glycosyl units found and the types of glycosidic linkages that compose them. Glycosides represent a major energy source for microorganisms, regardless of habitat (Flint et al. 2008, Larsbrink and McKee 2020, Ge et al. 2025). To deal with the structural diversity of glycosides, bacteria, like fungi (Barbi et al. 2021), have evolved a large array of transporters and enzymes to efficiently capture and metabolize them. Knowledge of the specificities and mechanisms of transporters is an important prerequisite for understanding how bacteria metabolize glycosides and thus colonize their habitats. This field is highly relevant to human and animal nutrition and health, as the type of bacteria that selectively ferment plant- and host-derived glycosides in the gut influences the host’s physiological functions and overall well-being (Koropatkin et al. 2012). Furthermore, from a biotechnological perspective, the robust processes that transform plant biomass into high-value products rely on efficient chassis strains with engineered transporters and metabolic networks (Lee et al. 2013, Lian et al. 2014). However, we only know the function of a small fraction of glycoside transporters. Even in the model organism *Escherichia coli*, ∼13% of the open reading frames encode membrane transport proteins, while around a quarter of them lack experimental validation (Ren and Paulsen 2007, Munro and Douglas 2022, Moore et al. 2024). Functional characterization is challenging because glycoside transporters are protein systems embedded in a membrane. They may also require other protein partners, either for substrate modification or to supply the energy necessary for the transport.

Advances in the development of culturing, sequencing and computing technologies have led to an increasing number of glycoside transporter sequences (Gelfand and Rodionov 2008), which are listed and classified in the Transporter Classification Database (TCDB, http://www.tcdb.org/) (Saier et al. 2021), together with transporters of monosaccharides, metals or amino acids. Based on sequence similarity, glycoside transporter sequences can indeed be assigned to one of four classes of transmembrane proteins, which have distinct mechanisms and architectures: ATP-binding cassette (ABC) transporters, major facilitator superfamily (MFS) transporters, phosphotransferase systems (PTS), and TonB-dependent transporters (TBDT), which, unlike the others, are found exclusively in Gram-negative bacteria because they are located in the outer membrane. Transporter specificity can be predicted based on similarity to previously characterized sequences. However, the limited number of characterized transporters and their often-low sequence similarity impair accurate prediction, which therefore relies on assignment to a superfamily or family based on the presence of specific motifs. Nevertheless, their specificity toward glycosides may vary dramatically, even among members of the same family (Diallinas 2014, Wang et al. 2020). The prediction of transporter specificity thus remains challenging.

The transport of glycosides in bacteria has been reviewed several times in recent years, each time with a specific focus on (i) the human gut (Cockburn and Koropatkin 2016, Schwalm and Groisman 2017); (ii) plant polysaccharides and prebiotics (Goh and Klaenhammer 2015); (iii) pathogenicity (Jeckelmann and Erni 2020); or (iv) the TBDT family of transporters (Grondin et al. 2017, Bolam and van den Berg 2018, Pollet et al. 2021, Silale and van den Berg 2023, Braun 2024). However, none of these studies synthesizes the current knowledge of experimentally validated glycoside transporters or addresses the challenges of their biochemical characterization. Here, we present the structural, taxonomic, and functional diversity of glycoside transporters in bacteria, with particular focus on the different methods used to characterize their functions, along with their advantages and their limits.

## Techniques for functional characterization of glycoside transporters

While advanced bioinformatics may help infer transporter specificity in the future, these approaches first require the collection of experimental evidence. A set of methods and techniques has emerged to identify the correct substrate for a given predicted glycoside transporter. We have classified them into three categories: analysis of growth phenotypes, determination of binding specificity, and determination of uptake specificity (Table 1).

**Table 1:** Comparison of the different methods used for functional characterization of glycoside transporters.

	Growth phenotype	Binding specificity	Kinetic parameters
**Approaches**	Growth assays in minimum medium supplemented by specific glycoside as sole carbon source	Affinity gel electrophoresis, pulldown assays Isothermal titration calorimetry Surface plasmon resonance Differential scanning fluorimetry (DSF), nanoDSF, Microscale thermophoresis Saturation transfer difference nuclear magnetic resonance High-performance anion-exchange chromatography coupled with pulsed amperometric detection (HPAEC-PAD)	Uptake experiments with radiolabeled or native glycosides
**Advantages**	Easily implemented	Easily determined and/or quantified	Transport ability quantified by radioactivity detection or solid-supported membrane-based electrophysiology or HPAEC-PAD quantification of glycosides
**Drawbacks**	Coupling to transcriptomics and/or genome engineering required due to functional redundancy	Limited to transport systems including soluble binding protein Transport specificity not necessarily related to binding	Limited to available radiolabeled glycosides Recombinant production of transmembrane protein or genome engineering required due to functional redundancy in native strains

### Analysis of growth phenotypes

The simplest method to screen a strain for its ability to internalize a glycoside, or one of its components, is to perform growth assays in minimum medium supplemented by a specific glycoside and monitor the turbidity of the culture medium. Glycoside utilization can also be detected easily using MacConkey indicator plates containing a solid differential medium designed to selectively isolate enteric Gram-negative bacteria (such as *E. coli*) and differentiate lactose-fermenting Gram-negative rods, as positive colonies turn red when they have been able to utilize lactose (Peng et al. 2009). Nevertheless, this approach is only applicable to a limited subset of Gram-negative bacteria, and they must be bile-resistant.

The choice of glycosides to be tested can be rationalized using genome sequencing to identify putative operons encoding for complete systems of glycoside sensing, binding, translocation across the cell membrane, and degradation by carbohydrate-active enzymes (CAZymes). The type of substrate targeted by a given operon can be predicted from the putative specificities of the CAZymes it encodes. Indeed, enzyme specificities are inferred from sequence homology with numerous biochemically characterized members of the same family, as annotated in the CAZy database (http://www.cazy.org) (Drula et al. 2022). Such genomic context analyses have, for instance, enabled the prediction of the specificity of three ABC transporters from *Thermus thermophilus* HB8 toward oligosaccharides in starch and β-glucans, with the targeted polysaccharides inferred from the distinct sets of CAZymes surrounding each transporter in the corresponding locus (Chandravanshi et al. 2019).

The upregulation of the transporter-encoding gene (or of the entire operon) during growth on a particular glycoside is a good indication that this transporter is involved in the utilization of this substrate. Quantification of gene expression levels requires transcriptomic analysis such as quantitative real-time PCR (qPCR) (Tauzin et al. 2016b). This technique is applicable when a limited number of genes is considered. For analysis at the genome scale, RNA chips (Martens et al. 2011) and DNA microarrays (Andersen et al. 2012) have been developed and have gradually been replaced by RNA sequencing (Despres et al. 2016). With improvements in sensitivity and decreasing costs, large-scale transcriptomic studies can be undertaken to compare the transcriptomes of thousands of bacteria (Lowe et al. 2017), giving indications of the specificity of putative transporters toward glycosides. However, transcriptomic analysis does not provide experimental proof of transporter specificity, for three reasons. First, transcript levels might not correlate well with protein levels (Liu et al. 2016). In this case, combining transcriptomics and proteomics can strengthen hypotheses regarding glycoside transporter function (Pichler et al. 2020, Martin et al. 2025). Second, the glycoside detected by the transcriptional regulator may differ from the glycoside translocated across the cell membrane (Schwalm and Groisman 2017). The gold standard for demonstrating that a transporter is responsible for the import of a glycoside is the observation of reduced or absent bacterial growth following the deletion or inactivation of the target gene (Webb et al. 2008, Andersen et al. 2012, Chen et al. 2015). Finally, functional redundancy is frequent in bacteria, where genes encoding several transporters can be upregulated and even overproduced during utilization of a complex glycoside (Kabisch et al. 2014). This functional redundancy may prevent the detection of growth phenotypes in the event of single gene deletion.

To avoid functional redundancy and facilitate genetic manipulation for gene inactivation or deletion, transporter function can also be characterized in recombinant strains.

For example, heterologous expression of the SusC/D gene pair from the inulin-using strain *Bacteroides thetaiotaomicron* 8736 in the non-inulin-using strain *B. thetaiotaomicron* VPI-5482 resulted in inulin use by the recipient strain, demonstrating that SusC/D from *Bacteroides thetaiotaomicron* 8736 is involved in inulin utilization (Joglekar et al. 2018). Furthermore, a locus encoding an ABC transporter and CAZymes from *Streptococcus mutans* LT11 gave *E. coli* the ability to ferment melibiose (Tao et al. 1993). Most recently, this approach has been extended to the study of transporters from uncultured bacteria (Tauzin et al. 2016b, Wang et al. 2020) in *E. coli*. By cloning various truncated forms of entire metagenomic loci encoding several transporters and at least one glycosidase into a fosmid, these studies demonstrated the specificity of each of these transporters toward oligosaccharides of various structures and lengths. Furthermore, Dagkesamanskaya et al. (2018) and Potocki-Veronese et al. (2023) developed workflows to perform recombinant *E. coli* growth assays in picoliter droplets using microfluidics, decreasing by several orders of magnitude the amount of substrate required to screen for transporter functionality and specificity.

Whether in native or recombinant strains, the ability of a bacterium to grow on glycosides and its efficiency in doing so are related to the levels of production and the combined efficiency of several proteins involved in sensing, binding, transporting, breaking down, and metabolizing the substrate. By analyzing the growth phenotype alone, it is impossible to quantify the efficiency of a transporter and, thus, to obtain a precise profile of its specificity toward glycosides of defined structures. This requires other methods, which are described below.

### Determination of binding specificity

For transporters involving binding proteins, known as substrate-binding proteins and surface glycan-binding proteins in ABC and SusC/D systems, respectively, it is easier to investigate the characteristics of the binding protein than those of the transmembrane protein. Binding proteins are soluble, which facilitates their recombinant production and analysis. Once the recombinant binding proteins have been successfully purified, different techniques are available to screen for their binding specificity:

- Affinity gel electrophoresis is a simple and rapid method of analyzing protein-binding affinities to soluble polysaccharides. It is based on the reduced mobility of proteins that interact with substrates (Tomme et al. 2000). Although small sugars, such as monosaccharides and disaccharides, cannot be used directly because they are freely mobile in the gels and have little effect on protein migration when bound, they can be used in a competition assay with a high-molecular-weight ligand, which allows the detection of binding sites for low-molecular-weight ligands.
- Insoluble polysaccharide pulldown assays can be used to test the binding affinity of SusC/D system surface glycan-binding proteins toward insoluble polysaccharides (Cockburn et al. 2016, Tamura et al. 2019). In this method, the insoluble polysaccharide is mixed with the protein. The unbound proteins remain in the supernatant, and the bound proteins are associated with the polysaccharide. The bound proteins can be released by adding an SDS buffer and boiling the samples. The different fractions can then be loaded onto an SDS-PAGE gel. Bound proteins can be quantified by comparing their concentration in the supernatant with that of the control.
- Isothermal titration calorimetry (ITC) is one of the most common methods for examining the binding specificity of glycoside-binding proteins. ITC allows one to determine the number of binding sites of a protein, the enthalpy of binding, and the association constant by measuring heat exchange during complex formation at a constant temperature (Bjelić and Jelesarov 2008, Leth et al. 2018, Bastos et al. 2023). This label-free method is suitable for measuring interactions with soluble polysaccharides and oligosaccharides. However, a significant limitation is the large quantity of soluble protein required for accurate measurements, particularly for proteins that are difficult to produce in large amounts.
- Surface plasmon resonance is also widely used to study binding kinetics, affinities, and stoichiometry (Cockburn et al. 2016, Theilmann et al. 2019, Pichler et al. 2020). This optical, label-free detection technique enables real-time monitoring of biomolecular interactions by measuring changes in the refractive index near a sensor surface. Typically, a protein is immobilized on the sensor surface and exposed to oligosaccharides in an aqueous phase. However, it is also possible to immobilize the oligosaccharides. This is particularly useful in surface plasmon resonance imaging, which enables the high-throughput screening of large numbers of oligosaccharides using carbohydrate microarrays (Karamanska et al. 2008, Nguyen et al. 2015). Only a few micrograms of protein are required.
- A simple way to screen for ligands is through a thermal shift assay, which is based on an increase in protein thermal stability when a ligand binds to it. Differential scanning fluorimetry (DSF) uses a standard quantitative polymerase chain reaction (qPCR) instrument to measure unfolding via selective dye fluorescence (Niesen et al. 2007, Boucher and Noll 2011, Hall 2019, Wu et al. 2023). NanoDSF relies on the intrinsic tryptophan fluorescence. Ligands that interact with a given protein can initially be ranked based on their Δ*T_m_*. However, these techniques rarely provide a dissociation constant at physiological temperatures (Grøftehauge et al. 2015). Steady-state tryptophan fluorescence spectroscopy measures the quenching of intrinsic tryptophan fluorescence upon ligand binding at a constant temperature (Phansopa et al. 2014). These last two techniques can be used for ligand screening of transmembrane domains because they are label-free (Sugihara et al. 2012). The requirement of detergent for membrane protein solubilization makes the traditional DSF assay challenging for these proteins. Microscale thermophoresis relies on either the intrinsic tryptophan fluorescence or a covalently attached fluorophore. However, it measures the differential thermophoretic movement of biomolecules in free and bound states through a temperature gradient (Santiveri et al. 2017, Correia et al. 2021). These spectroscopic methods require a small amount of protein and are relatively easy to set up. However, precise quantification of affinities requires thoughtful data analysis or alternative biophysical techniques.
- Saturation transfer difference nuclear magnetic resonance (STD-NMR) is a versatile, label-free method for screening ligands and determining binding affinities (Bell et al. 2019). Remarkably, it can be used to study membrane proteins (Hall et al. 2020) and distinguish transported sugars from sugar inhibitors (Ahn et al. 2026). STD-NMR is a powerful method that provides structural details of the interaction by identifying ligand contacts with the protein in the complex (Monaco et al. 2017).

Finally, all of these techniques require purified proteins, necessitating the optimization of recombinant protein production. It is important to note that the binding specificity of these proteins do not permit the direct determination of uptake specificity. For instance, MalE, the SBP of the maltose ABC transporter from *E. coli* K-12, binds to α- and β-cyclodextrins; however, these substrates cannot be taken up by the maltose ABC transporter (Quiocho et al. 1997, Mächtel et al. 2019). Wang et al. (2020) developed a simple method to quantitatively analyze the binding specificity of the overall transporter, regardless of its family. This method is based on the use of crude cell debris and intact cells (native or recombinant), and nonlabeled substrates. Very small concentration variations of these substrates (less than one nanomole) are monitored by high-performance anion-exchange chromatography coupled with pulsed amperometric detection. This method not only enables binding quantification, but also determines uptake specificity.

### Determination of kinetic parameters

As explained previously, in native strains, the multiplicity of carbohydrate transporters can bias the results of specificity screening. To solve this problem, the targeted transporter can be expressed in recombinant strains lacking the function of interest (Wei et al. 2012), or in membrane vesicles or proteoliposomes (Cao et al. 2011, McCoy et al. 2016). The isolation of a transporter within proteoliposomes enables the determination of its kinetic parameters using radioactive substrates (Drew et al. 2021). However, it requires a dedicated secure environment for handling radiolabeled compounds. In addition, most glycosides are not available in radioactive form or are prohibitively expensive, which precludes large-scale screening campaigns. However, other techniques, such as solid-supported membrane-based electrophysiology, can be used instead (Grewer et al. 2013, Bazzone et al. 2022, Dong et al. 2024). Nevertheless, these approaches may fail when the transport proteins are not active due to issues with membrane targeting, insertion or folding during the purification or reconstitution process (Majd et al. 2018). These approaches can be even more limiting for TBDTs, as they require interaction with the TonB protein and the associated proton motive force to be functional. To circumvent these limitations, the highly generic method described earlier was recently developed to quantify transporter uptake rates (Wang et al. 2020).

Less generic methods also exist, focusing on particular transport mechanisms involving energy-coupling or substrate modification. Phosphoenolpyruvate (PEP)-dependent glycoside phosphorylation, which is a specific feature of PTS transporters, can be tracked by analyzing the amount of pyruvate in cell-free systems, to deduce the amount of phosphorylated glycosides (Martin and Russell 1987). The amount of pyruvate can be monitored by quantifying the product of NADH oxidation by lactate dehydrogenase. For ABC transporters, glycoside uptake requires energy from ATP-binding and hydrolysis. Thus, the specificity of ABC transporters can be determined by testing growth on the target substrate when ATP synthesis is inhibited with an inhibitor such as iodoacetate (Kaplan and Hutkins 2003).

## TonB-dependent transporters and associated surface glycan-binding proteins

### The SUS paradigm

**Figure 1:**
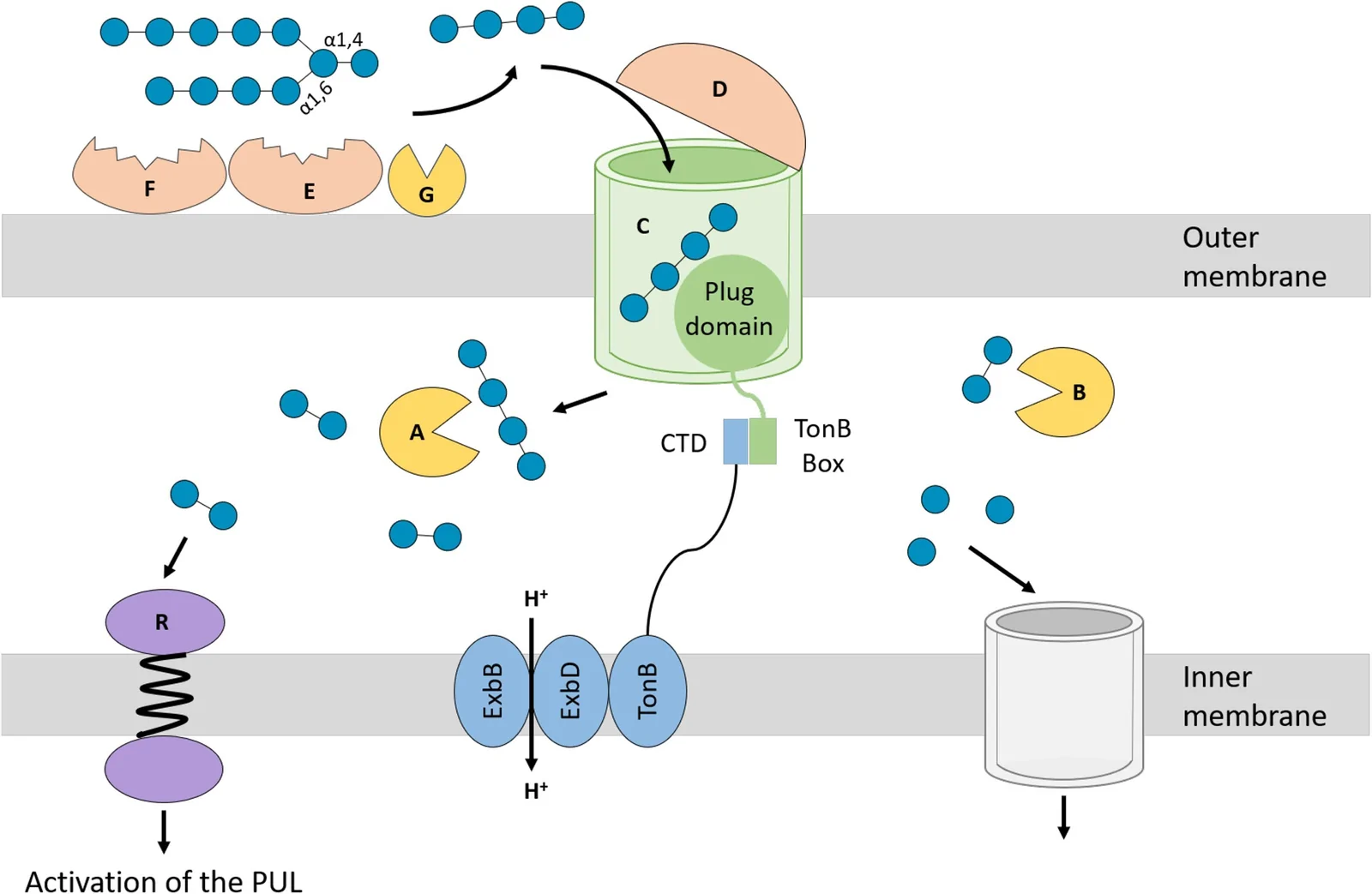
**Schematic representation of the starch utilization system in *Bacteroides thetaiotaomicron***. Starch is bound to the cell surface by starch-binding outer membrane lipoproteins SusDEF. Subsequent hydrolysis by the membrane-tethered α-amylase SusG generates oligosaccharides small enough to pass through the TonB-dependent transporter SusC. Once in the periplasm, the neopullulanase SusA and the α-glucosidase SusB process the oligosaccharides into glucose. The monosaccharide is then transported into the cytoplasm by an unknown transporter. CTD: C-terminal domain.

A defining feature of Gram-negative bacteria is their dual membrane structure, consisting of an outer membrane and an inner membrane separated by the periplasm. Therefore, for a glycoside to be imported, it must necessarily pass through the outer membrane. In the late 1980s, the Salyers’ group identified and characterized a locus involved in starch utilization in *Bacteroides thetaiotaomicron* 5482 (Tancula et al. 1992) and later named the starch utilization system (SUS) (D’Elia and Salyers 1996). This became the prototype of the polysaccharide utilization loci (PUL) concept (Bjursell et al. 2006). In this strain, the SUS locus is composed of eight genes named *susRABCDEFG* (Fig. 1). At the cell surface, starch recognition and binding are mediated by outer membrane binding proteins SusD to SusF. Initial degradation into maltooligosaccharides is performed by the surface lipoprotein SusG, an α-amylase (Shipman et al. 1999). The oligosaccharides are internalized via the TonB-dependent transporter (TBDT, SusC) (Reeves et al. 1996) before being subsequently degraded by periplasmic glycoside hydrolases (SusA and SusB) (Smith and Salyers 1991, D’Elia and Salyers 1996). Finally, SusR, an inner membrane-spanning sensor/regulator protein, recognizes periplasmic maltose and triggers upregulation of the SUS locus (D’Elia and Salyers 1996). PULs can display other regulator types, including hybrid two-component systems, or extracytoplasmic sigma factor/anti-sigma factors (Ravcheev et al. 2013). In addition, post-transcriptional regulation involving small noncoding RNAs and RbpB, an RNA-binding protein, has recently been described in *B. thetaiotaomicron* (Rüttiger et al. 2025).

Both SusC and SusD have been identified as the main contributors to substrate uptake, while SusE and SusF, which are not present in all PULs, play auxiliary roles in substrate recognition and binding. The SUS paradigm and the PUL concept have been extended in recent decades to other polysaccharide utilization pathways in Bacteroidota (formerly Bacteroidetes). Tandem *SusD*-like and *SusC*-like genes are considered hallmarks of PULs (Bjursell et al. 2006), and the presence of these *SusC/D*-like gene pairs is used to identify PULs in Bacteroidota genomes (PULDB; https://www.cazy.org/PULDB/) (Terrapon et al. 2015). Nevertheless, the characterization of a non-canonical PUL and genes distal to the PUL, including a *SusC/D*-like pair (Ficko-Blean et al. 2017), strengthens the proposition that the definition of a PUL should not be restricted to the presence of a *SusC/D*-like pair (Grondin et al. 2017). Consequently, prediction of CAZyme clusters, devoid of *SusC/D*-like pairs, have been included in PULDB (Terrapon et al. 2018).

### Functional diversity of SUS-like systems

In the past decade, 35 Bacteroidota SUS-like systems have been characterized in detail (Supplementary Table 1), targeting a large diversity of polysaccharides, including xyloglucan (Larsbrink et al. 2014, Tauzin et al. 2016a, Foley et al. 2019), xylans (Rogowski et al. 2015), mannans (Cuskin et al. 2015), β-glucans (Tamura et al. 2017, Temple et al. 2017, 2019, Déjean et al. 2020, 2021, Golisch et al. 2024, Li et al. 2024), chitin (Larsbrink et al. 2016), galactomannan (Bågenholm et al. 2017, 2019), arabinogalactans (Cartmell et al. 2018), pectins (Ndeh et al. 2017, Luis et al. 2018), levan (Sonnenburg et al. 2010), porphyran (Hehemann et al. 2012), carrageenan (Ficko-Blean et al. 2017), glycosaminoglycans (GAGs) (Cartmell et al. 2017, Ndeh et al. 2020), and mucins (Crouch et al. 2020, Luis et al. 2021). Some β-glucan activities have been highlighted in PULs from rumen uncultured Bacteroidota (Pope et al. 2012, Naas et al. 2014, Mackenzie et al. 2015), and the SusD-like proteins of these PULs were shown to bind strongly to Avicel and filter paper (Mackenzie et al. 2012, Naas et al. 2014). Since growth studies and transcriptomic analyses of these metagenomic species have been impossible to perform to date, there is no evidence that the native strains containing these PULs are really able to utilize cellulose.

The complexity of PUL organization reflects the structural complexity of their cognate substrate, with the presence of genes encoding enzymes that are not CAZymes, such as sulfatases, peptidases, epimerases, and members of the ROK (Repressors, ORFs, and Kinases) family, as well as additional transporters such as MFS transporters (Terrapon et al. 2018). For example, the utilization of yeast α-mannan by *Bacteroides ovatus* is mediated by three PULs working in synergy and encoding more than a dozen glycoside hydrolases, a few phosphatases and three SusC/D-like transport systems (Cuskin et al. 2015). Despite a similar level of complexity, xyloglucan is degraded by *B. ovatus* through a single PUL consisting of 12 genes, including eight glycoside hydrolases and a single SusC/D-like pair (Larsbrink et al. 2014). Surprisingly, a PUL from *Flavobacterium johnsoniae* enabling conversion of chitin, a simpler polysaccharide in terms of monosaccharide composition and branching, contains only four enzymes but two SusC/D-like pairs (Larsbrink et al. 2016). Both SusD-like proteins were shown to bind crystalline chitin and genetic engineering of the strain revealed functional overlap between the two SusC/D pairs. Understanding why a PUL targeting a polysaccharide only made by a repetition of the same unit (*N*-acetylglucosamine) contains two SusC/D pairs, whereas a xyloglucan PUL contains only one, requires further investigation. There is likely a functional role at the cellular level that remains to be discovered.

Until now, the main focus of the biochemical characterization of PULs has been on glycoside hydrolases and surface glycan-binding proteins. The characterization of the transmembrane component of SUS systems, the SusC, is more difficult.

### Structure and mechanism of TonB-dependent transporters and associated surface glycan-binding proteins

SusC is a TBDT structurally characterized by an N-terminal plug domain inserted into a 22-stranded β-barrel. It allows oligosaccharides to be actively transported from the cell surface into the periplasm. However, no energy source is known for the outer membrane. Instead, the energy is provided by the interaction with the TonB protein, which is located in the cytoplasmic membrane (Fig. 1). The TonB protein contains an N-terminal α-helix that anchors it in the inner membrane, an intrinsically disordered linker spanning the periplasm and a C-terminal globular domain that binds to TBDTs (Krewulak and Vogel 2011). The binding of the substrate by the TBDT induces an allosteric rearrangement of the plug domain, which contains a conserved binding motif (TonB box). This motif is then released into the periplasmic space. There, the TonB box interacts with the C-terminal domain of TonB, while the N-terminus of TonB forms a complex with the inner membrane proteins ExbB and ExbD (the TonB complex). This system uses the proton motive force of the inner membrane to move the plug and create a pathway through the TBDT (Larsen et al. 1999, Wang et al. 2023, Zinke et al. 2024). Gene deletion studies have recently shown that two of the 11 TonB-encoding genes in *B. thetaiotaomicron* may be functionally redundant, allowing proper growth on starch (Pollet et al. 2023).

SusD-like proteins are cell-surface glycan-binding proteins involved in the capture of extracellular glycans. Interestingly, SusD-like proteins share no sequence similarity with known carbohydrate-binding modules and are notably around twice their size (around 65 kDa) (Foley et al. 2016). In 2008, the first crystal structure of a SusD from *B. thetaiotaomicron* VPI-5482 involved in starch utilization was solved (Koropatkin et al. 2008). Thus far, the overall structure of SusD-like proteins has been shown to consist of a region of α-helices organized into four tetratricopeptide repeat units and a more variable region containing numerous loops and short α-helices. The variable region is unique to each SusD-like protein. It depends on cognate substrate and includes an optimized platform for specific substrate binding, mainly provided by at least two tryptophan residues. SusD-like proteins are attached to the outer membrane via a lipidated N-terminal cysteine projecting the protein above the membrane surface to facilitate glycan capture (Koropatkin et al. 2008, Cameron et al. 2014). In rare instances, some SusD-like proteins do not efficiently bind the cognate substrate of the transporter. In such cases, substrate binding is ensured by additional surface glycan-binding proteins encoded by the same PUL or elsewhere in the genome (Luis et al. 2018, Déjean et al. 2020). To our knowledge, there is only one example of a non-binding SusD-like protein present in a PUL that does not encode additional surface glycan-binding proteins (Tauzin et al. 2022). Notably, this PUL is involved in xylooligosaccharide utilization by an uncultured human gut *Bacteroides* strain, and this work highlights that the term “surface glycan-binding proteins”, generally used for SusD-like proteins, is not universally applicable.

**Figure 2:**
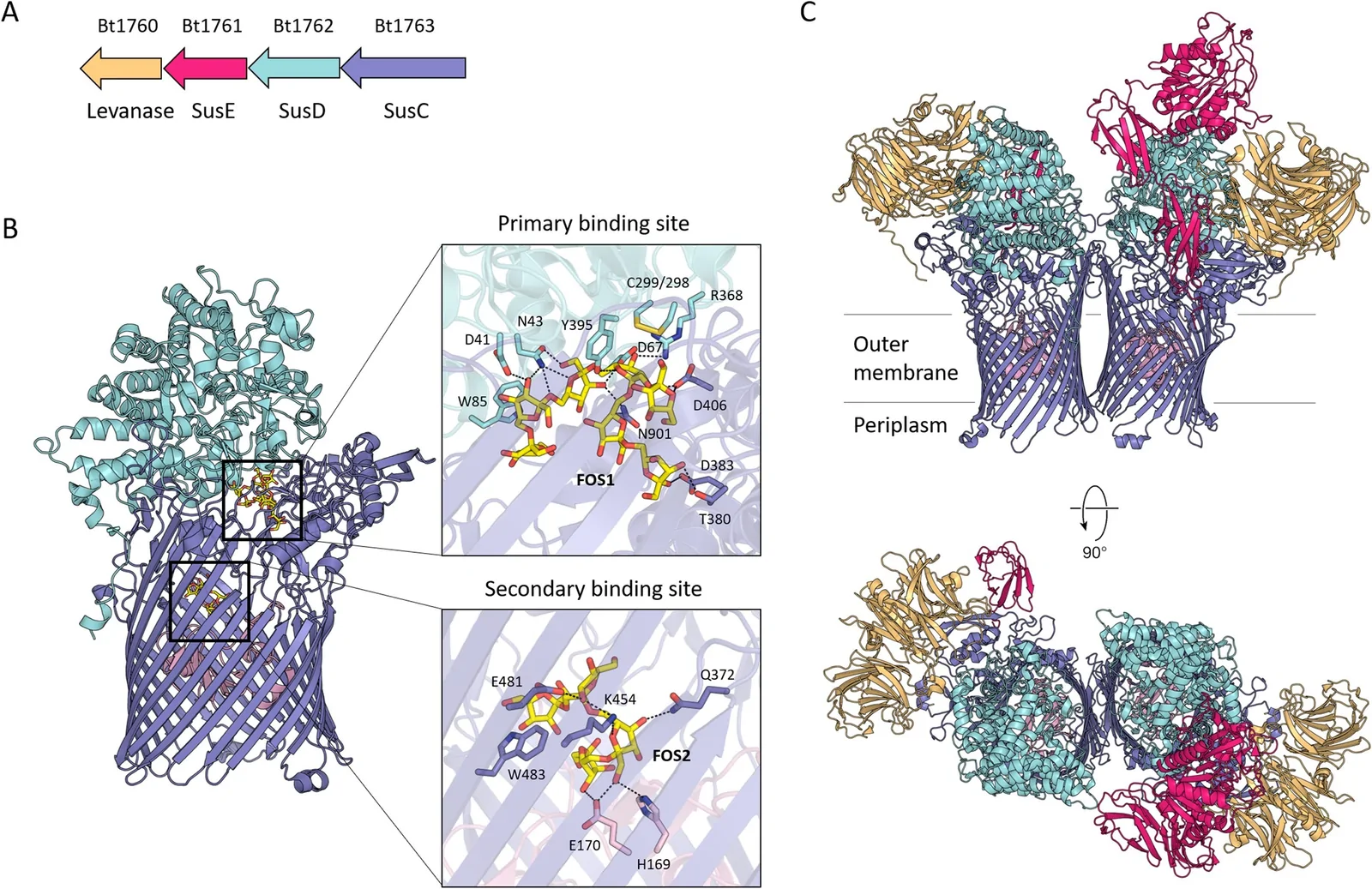
**Substrate recognition and spatial organization of the levan PUL in *Bacteroides thetaiotaomicron***. A) Gene organization of the levan PUL. SusE is a surface glycan-binding protein. B) The SusCD transporter in the closed state, with two fructo-oligosaccharides (PDB ID: 6ZAZ). The SusC plug-domain is colored pink. The two panels illustrate the detailed interactions between the fructo-oligosaccharides (FOS) and the residues in SusD and SusC. The FOS are shown as sticks, with carbon atoms colored yellow and oxygen atoms colored red. FOS1 contains seven fructose units, including one branched fructose unit. FOS2 contains four fructose units. Protein–oligosaccharide interactions are marked by dotted lines connecting the sugars and residues. C) The levan utilisome forms homodimers. The data were generated by cryo-electron microscopy (map EMD-1529), but only the fitted models are shown (PDB ID 8AA2).

In SUS, SusC physically interacts with SusD to form a complex that represents a minimal system for starch utilization by *B. thetaiotaomicron* VPI-5482 when complemented with SusG (Cho and Salyers 2001). However, studies using non-binding SusD-like mutants showed that substrate binding to SusD-like proteins is not a prerequisite for growth, since expression of other surface glycan-binding proteins (SusE and SusF-like proteins from the same PUL) tends to compensate for this loss (Tauzin et al. 2016a, Cameron et al. 2014). Deletion of SusD-like proteins impairs growth on the cognate substrate, implying that these proteins play an essential structural role in SusC/D pair formation in some systems, where binding ability is also not a prerequisite for substrate transport (Koropatkin et al. 2008, Tauzin et al. 2016b). By contrast, it was recently demonstrated that both substrate binding and the physical presence of the SusD-like protein are crucial for efficient utilization of mixed-linkage glucans by *B. ovatus* (Tamura et al. 2019). To date, only three 3D structures of three distinct SusC/D complexes have been solved. In Glenwright et al. (2017), the authors used X-ray crystallography and molecular dynamics to determine the structure of two SusC/D pairs from *B. thetaiotaomicron* VPI-5482. One pair (BT2263-64) is found in a four-component complex of unknown function, while the second (BT1762-63) is part of a levan PUL (Sonnenburg et al. 2010). Glenwright et al. suggested a pedal-bin mechanism for nutrient acquisition by SusC/D-like systems, with SusD forming the lid and SusC the bin. In the absence of the ligand, SusD moves away from SusC in a hinge-like fashion, exposing the substrate-binding site to the extracellular environment. After substrate binding, the SusD ‘lid’ swings into a closed state, resulting in stabilization of the complex through interaction between the ligand and both SusC and SusD. The substrate then moves into the SusC ‘bin’, preceding binding of TonB to the TonB box and subsequent conformational changes leading to substrate release into the cell. The structure of SusD is not altered in the closed state and interaction with SusC involves the highly variable region forming the substrate-binding site of the SusD-like proteins. According to the authors, this pedal-bin mechanism is consistent with envelopment of the whole ligand molecule, which precludes the import of undigested high-molecular-weight glycans. Interestingly, SusC/D is dimeric, with the dimer interface formed by SusC. Cryo-EM studies on the same complex confirmed the dimeric nature of the transporter and revealed three distinct states—open–open, open–closed, and closed–closed—based on the position of the SusD lid (Gray et al. 2021). Recently, cryo-EM experiments have also enabled researchers to solve the 3D structure of the four components (SusC, SusD, SusE, and enzyme) of the levan (BT1760-63) and dextran (BT3087-90) PULs (Fig. 2). The SusC/Ds remain dimeric, forming octameric complexes in which the positioning of the subunits is consistent with pedal-bin transport (White et al. 2023). In this case, the authors identified two binding sites within the SusC barrel: one at the interface of SusC and SusD, which binds a hexasaccharide, and a second at the bottom of the SusC cavity, where a tetrasaccharide is also in contact with the plug. Interestingly, at higher threshold levels, diffuse density was detected between the two binding sites, indicating multiple conformations and the absence of interaction with SusC in this segment. These observations suggest that the SusC/D core is promiscuous and can accommodate relatively long oligosaccharides (probably up to a degree of polymerization (DP) of 15). The ability of SusC/D transporters to import large oligosaccharides is particularly evident in the degradation of yeast α-mannan by the human gut bacterium *B. thetaiotaomicron* (Cuskin et al. 2015). In fact, surface enzymes have a limited effect on the polysaccharide, resulting in the formation of large oligosaccharides. These are imported by the SusC/D transporters located on the outer membrane and are subsequently depolymerized to mannose by the action of periplasmic enzymes. Co-culture experiments have shown that this strategy allows for the selfish utilization of this polysaccharide. Thus, the characteristics of PULs make the Bacteroidota specialists in the degradation of high-molecular-weight organic matter. It is noteworthy that SusC/D pair cannot always be linked with glycan utilization, as many pairs appear isolated in the genome or surrounded by proteins of unknown function (Bolam and van den Berg 2018, Lapébie et al. 2019). However, as discussed above, an isolated SusC/D pair could also function in synergy with other PULs to achieve complete degradation of a complex polysaccharide, such as pectin by *B. thetaiotaomicron*, where one of the three PULs includes only a SusC/D pair (RG-II PUL2) (Ndeh et al. 2017).

### Taxonomical diversity of TonB-dependent transporters

Since the TonB protein is exclusively found in Gram-negative bacteria, it follows that TBDTs can only occur in these organisms (Williams-Jones et al. 2025). The presence of PULs is a unique feature of members of the Gram-negative phylum Bacteroidota. The SusC/D pair is used as a hallmark for PUL identification, and PULDB lists 81 235 SusC/D-containing PULs from 2494 Bacteroidota species (July 2025 release). However, TonB-dependent glycoside transporters are also present in Gammaproteobacteria, such as in the plant pathogen *Xanthomonas campestris* pv. campestris (Blanvillain et al. 2007, Déjean et al. 2013). They are also identified in sizable operons found in several marine Gammaproteobacteria targeting macroalgal polysaccharides (Schultz-Johansen et al. 2018, Hettle et al. 2019). Until recently, Gammaproteobacteria were thought to lack SusD-like proteins, but recent work reported the identification of a SusD-like protein and a SusC-like TBDT in a fructan polysaccharide utilization locus of the marine bacterium *Pseudoalteromonas distincta* (Zühlke et al. 2026). Phylogenetic analyses revealed that SusC/D-like proteins are present in other Gammaproteobacteria, where they are consistently associated with fructan PULs and likely acquired by lateral transfer from Bacteroidota.

Finally, the PUL concept has further been extended to Gram-positive bacteria belonging to the other dominant phylum, Bacillota (Sheridan et al. 2016). As Gram-positive bacteria do not possess an outer membrane, unlike Gram-negative bacteria, they lack SusC/D-like transporters. A new definition of PULs has therefore been established for these organisms, in which Gram-positive PULs (gpPULs) denote bacterial loci encoding for at least one glycoside-degrading enzyme, one transport system, and one transcriptional regulator.

## ABC transporters

ABCs form one of the largest transport systems. ABCs are present in many eukaryotes and prokaryotes, and even in some large viruses (Ford and Beis 2019). These permeases are generally primary active transporters, which may act either as importers or exporters depending on the direction of cargo molecule translocation (Locher 2016). They are able to transport various substrates in response to ATP hydrolysis (Mächtel et al. 2019). All ABC transporters share a core architecture comprising two transmembrane domains (TMDs) and two cytosolic nucleotide-binding domains (NBDs), where ATP binds and is hydrolyzed (Beis 2015). Glycoside importers also require cooperation with an additional cognate partner: a substrate-binding protein, also named substrate-specific binding component (both abbreviated SBP) (Theodoulou and Kerr 2015, Wilkens 2015). However, exceptions exist in a subgroup of ABC transporters lacking SBPs, involved in the uptake of vitamins and micronutrients and referred to as energy-coupling factor (ECF) transporters (Erkens et al. 2012). Recently, a sucrose ABC importer lacking an SBP has been described (Wang et al. 2020), but its mechanism remains to be determined. A recent publication brings together numerous authors advocating for a new ABC transporter classification based on structural homology of the TMDs (Thomas et al. 2020).

### Diversity of sequences and structures

Among the ABC importers currently listed in the TCDB, carbohydrate-specific transporters are clustered within two families: carbohydrate uptake transporter-1 (CUT1 annotated with TC# 3.A.1.1) and carbohydrate uptake transporter-2 (CUT2 annotated with TC# 3.A.1.2) (Saier 2000). Members of the CUT1 family are specific to a variety of oligosaccharides with a degree of polymerization ranging from 2 to 7, as well as polyols, glycerol and glycerol phosphate. Members of the CUT2 family transport only monosaccharides (Schneider 2001). However, this does not exclude the existence of exceptions, such as a d-xylose and l-arabinose ABC transporter in *Haloferax volcanii* H26 belonging to the CUT1 family (Johnsen et al. 2019). In addition, members of the peptide/opine/nickel uptake transporter (PepT) family (3.A.1.5) were also found to be involved in glycoside transport (Conners et al. 2005, Nanavati et al. 2006, Andersen et al. 2012). It can therefore be expected that the number of families containing glycoside transporters will increase as new transport specificities are discovered.

All these characterized glycoside transporters are SBP-dependent. Generally speaking, CUT1 ABC transporters contain two separate transmembrane proteins interacting with a protein carrying two copies of the NBDs (Schneider 2001). In glycoside ABC transporters of the PepT family, either the two TMDs or the two NBDs are located in separate proteins.

The selectivity and specificity of carbohydrate ABC transporters are largely determined by the SBPs, which are the most diverse components of the ABC systems, whereas the NBDs and, to a lesser extent, the TMDs are clearly more similar. Indeed, SBPs have relatively low sequence similarity (less than 20%), and phylogenetic analyses based on multiple sequence alignments did not yield a stable tree (Berntsson et al. 2010). Traditionally, SBPs have been categorized into different groups based on their substrate specificity, with glycoside ABC transporters clustered within the CUT1 families (Tam and Saier 1993). In 2010, SBPs of ABC importers were reclassified into six clusters based on their available three-dimensional structures. SBPs binding glycosides are spread across three different clusters (B, C, and D) (Berntsson et al. 2010). A seventh cluster was proposed to extend the structural classification, as the structure of the fructooligosaccharide-binding protein of an ABC transporter displays additional structural elements defining a larger ligand-binding cavity relative to the canonical SBPs, as well as an EF hand-like calcium-binding site (Culurgioni et al. 2017).

### Functional diversity

Since the term ‘ABC transporters’ was defined in 1990, a huge number of proteins have been annotated as ABC transporters, as described in the following section (Holland 2019). However, only a small set of ABC transporters have been experimentally proven to be involved in glycoside utilization by bacteria, using one or more of the methods described earlier (Supplementary Table 2). In some cases, only the function and structure of SBPs were characterized because these proteins are highly soluble and thus easier to characterize than TMDs. However, substrate specificity determined by binding assays does not always correspond to actual transport specificity. The MalE SBP of the maltose ABC transporter from *E. coli* K-12 binds α- and β-cyclodextrins, whereas these substrates cannot be taken up by the maltose ABC transporter (Quiocho et al. 1997). Yet, in a few cases, the ABC transporters—such as those targeting maltooligosaccharides and alginate—were characterized in detail by combining biochemical, molecular and structural approaches (Maruyama et al. 2019, Mächtel et al. 2019).

### Taxonomical diversity

We examined the taxonomical diversity of all characterized bacterial ABC transporters targeting glycosides from the CUT1 and PepT families (Table 2). These 124 glycoside ABC transporters occur widely in bacteria, including seven phyla and 21 orders from different environments, some of which include human pathogens such as *Streptococcus mutans* and *Enterococcus faecalis* (Webb et al. 2008, Sauvageot et al. 2017). Of the 124 glycoside ABC transporters, more than 90% were assigned to the four phyla Bacillota, Actinomycetota, Pseudomonadota, and Thermotogae. However, the actual taxonomical distribution of glycoside ABC transporters is highly biased because the sequencing and biochemical characterization efforts provide unequal coverage of bacterial diversity. A search of the Pfam 32.0 database for the domain (PF01547) of the SBP of the maltose ABC transporter alone (El-Gebali et al. 2019) retrieved 25 735 sequences across 4106 species, highlighting both the huge diversity of ABC transporters targeting glycosides and the fact that extremely few of them have been characterized (Supplementary Table 2). Unlike other phyla, the Bacteroidota phylum has few ABC transporters. In the Pfam 32.0 database, 56 Bacteroidota species harbor 74 sequences containing the Pfam domain of the SBP of the maltose ABC transporter, and we did not find any characterized in the literature. This low representation suggests that in this phylum, they play a minor role in the import of small carbohydrates from the periplasm to the cytoplasm (Barbeyron et al. 2016, Martin et al. 2025). More generally, the taxonomical diversity of the glycoside-targeting ABC transporters characterized to date is far from an accurate representation, as most ABC transporters remain uncharacterized.

**Table 2:** Phylum and order distribution of the characterized glycoside-targeting ABC transporters in bacteria.

Phylum	Order	Number of characterized ABCs	Percentage of ABCs characterized	
			Order	Phylum
Actinomycetota	Bifidobacteriales	32	24.43	37.4
	Corynebacteriales	6	4.58	
	Micrococcales	1	0.76	
	Kitasatosporales	10	7.63	
Bacillota	Bacillales	13	9.92	46.55
	Bacteroidales	1	0.76	
	Eubacteriales	22	16.79	
	Lactobacillales	8	6.11	
	Thermoanaerobacterales	16	12.21	
	Paenibacillales	1	0.76	
Pseudomonadota	Enterobacterales	2	1.53	4.58
	Hyphomicrobiales	2	1.53	
	Sphingomonadales	1	0.76	
	Bdellovibrionales	1	0.76	
Cyanobacteria	Synechococcales	1	0.76	1.52
	Nostocales	1	0.76	
Thermotogae	Thermotogales	4	3.05	3.05
Deinococcus–Thermus	Thermales	6	4.58	4.58
Campylobacterota	Campylobacterales	1	0.76	0.76
Fusobacteria	Fusobacteriales	1	0.76	0.76
Aquificota	Aquificales	1	0.76	0.76

**Table 3:** Phylum and order distribution of the characterized glycoside-targeting PTS transporters in bacteria.

Phylum	Order	Number of characterized PTSs	Percentage of PTSs characterized	
			Order	Phylum
Bacillota	Bacillales	8	12.5	76.56
	Lactobacillales	38	59.38	
	Clostridiales	2	3.12	
	Thermoanaerobacterales	1	1.56	
Pseudomonadota	Enterobacterales	10	15.63	18.75
	Vibrionales	1	1.56	
	Orbales	1	1.56	
Actinomycetota	Corynebacteriales	2	3.13	4.69
	Kitasatosporales	1	1.56	

### Mechanism of glycoside import by ABC transporters

**Figure 3:**
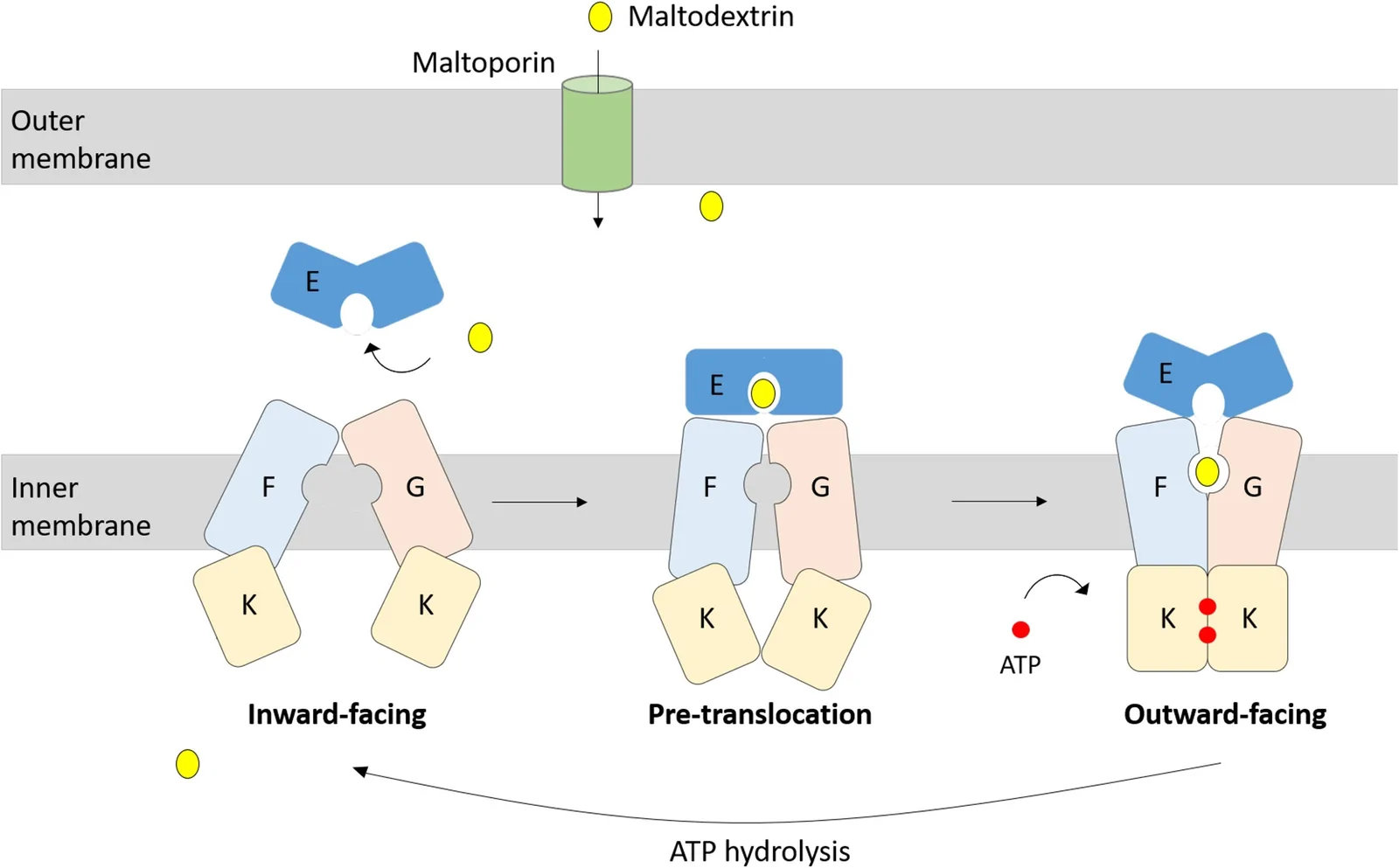
**Uptake of maltooligosaccharides by the MalFGK2 ABC transporter in Gram-negative bacteria**. These molecules diffuse across the outer membrane through a porin, bind to the periplasmic substrate-binding protein MalE and are then transported across the inner membrane by the MalFGK2 ABC transporter. The MalF and MalG proteins each have two transmembrane domains, and the MalK protein, which is a cytosolic nucleotide-binding domain, forms a homodimer to hydrolyze ATP.

Since the sequencing of the genes encoding the maltose ABC transporter in the 1970s (Silhavy et al. 1979), the functional and structural characterization of maltooligosaccharide transporters, especially in *E. coli*, has established a glycoside ABC transporter paradigm. Maltooligosaccharide import occurs via a classic alternating-access mechanism and substrate specificity is controlled by the outer membrane maltoporin, the periplasmic protein MalE, and the transmembrane subunits (MalFG) (Fig. 3) (Schneider 2001, Mächtel et al. 2019).

**Figure 4:**
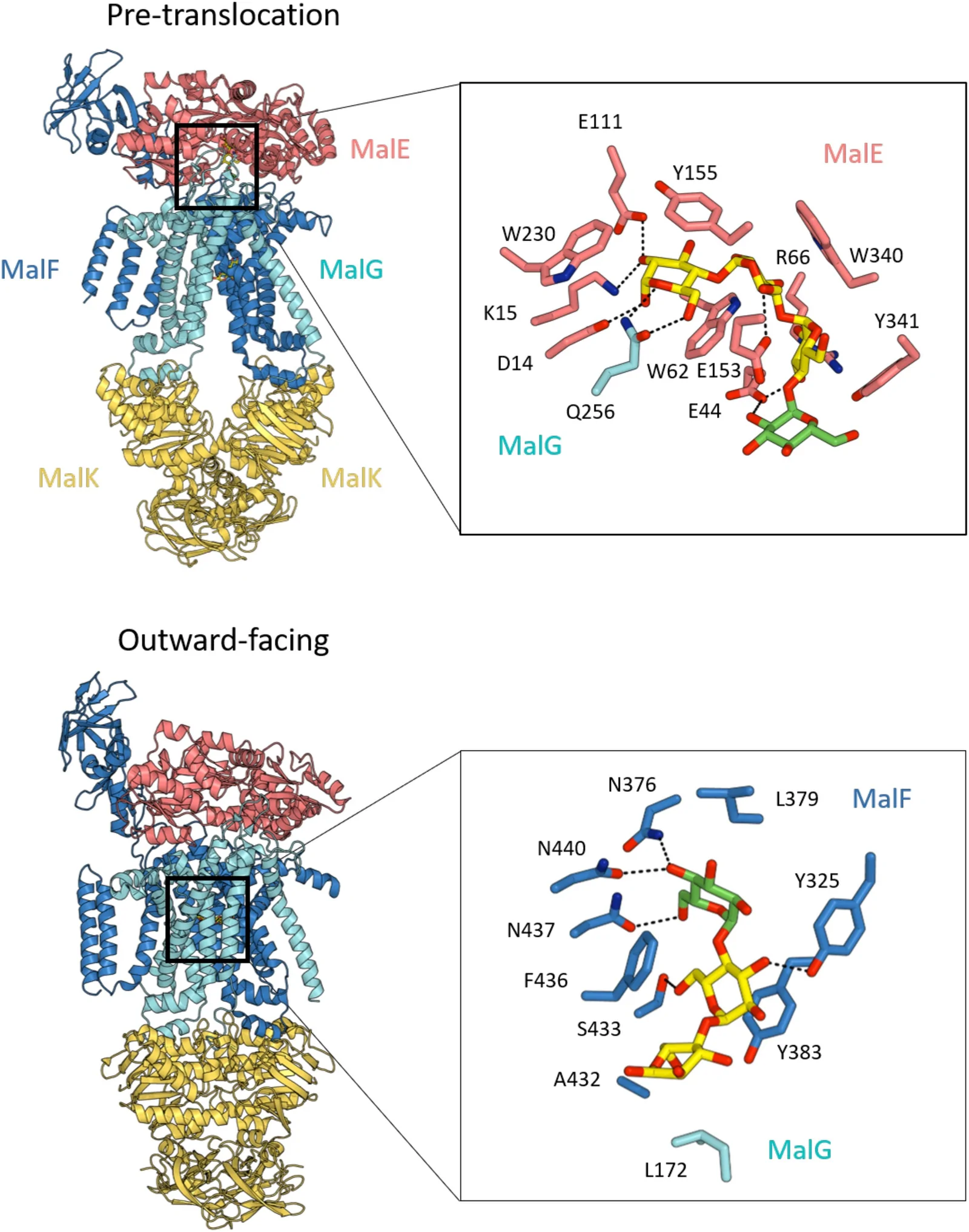
**Structure of the *E. coli* maltose ABC transporter in complex with maltodextrins**. The pretranslocation (PDB ID: 4KHZ) and outward-facing (PDB ID: 4KI0) states are shown with zoomed-in views of the amino acids that interact with the oligosaccharides. MalE and MalG interact with the glucosyl units from the reducing end. MalF and MalG interact with the glucosyl units from the non-reducing end. The glucose units are shown as sticks, with carbon atoms colored yellow and oxygen atoms colored red. The glucose molecule with green carbon atoms indicates the non-reducing end of each oligosaccharide. Protein–oligosaccharide interactions are marked as dotted lines between the sugars and the residues.

MalE consists of two globular lobes (the N-terminal and C-terminal domains), between which a substrate-binding pocket is formed. The shape complementarity, extensive hydrogen bonding, and aromatic stacking interactions together account for the high-affinity binding (in the micromolar range) of MalE to maltooligosaccharides (Quiocho et al. 1997) (Fig. 4). In the absence of a ligand, MalE predominantly adopts an open conformation. Once the maltooligosaccharide passes through the maltoporin, MalE completely traps the substrate at the interface of the two lobes and then docks with the transmembrane subunits, triggering ATPase activity by the MalK homodimer and initiating the transport cycle (Bordignon et al. 2010, Mächtel et al. 2019). Crystal structures of the MalE-MalFGK2 transporter complex from *E. coli* K-12 bound to a maltooligosaccharide in two different conformational states revealed that i) in the pre-translocation structure, MalG forms two hydrogen bonds with the maltooligosaccharide involving up to four glucosyl units at the reducing end, and ii) in the outward-facing conformation, the MalF transmembrane subunit binds glucosyl units from the non-reducing end (Oldham et al. 2007, 2013). Thus, MalFG provides another level of substrate selectivity, beyond that conferred by MalE. The substrate can be imported unidirectionally, with TMDs switching between inward- and outward-facing conformations. ATP hydrolysis is stimulated both by MalE, which stabilizes the semi-open configuration of MalFGK2, and by open MalE, which stabilizes the closed state of MalFGK2 (Mächtel et al. 2019). The binding cavity of the pre-translocation state of the MalE-MalFGK2 complex is large enough to accommodate seven glucosyl units, corresponding to the maximum DP of the oligosaccharides that can be transported (Oldham et al. 2013). The maltooligosaccharide ABC transporter could serve as a valuable model for understanding how other glycoside ABC transporters evolve and function, despite their structural and functional diversity.

## PTS transporters

**Figure 5:**
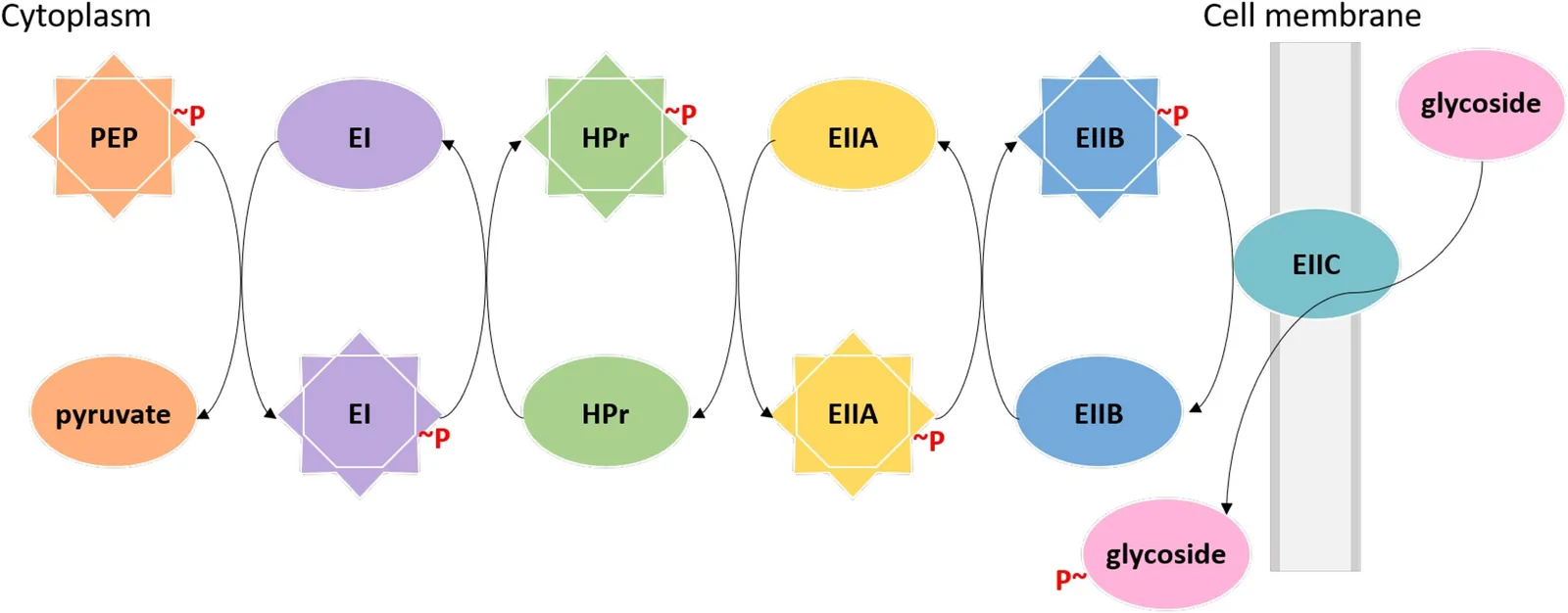
**Schematic illustration of the process of glycoside transport and phosphorylation by the PTS**. The figure shows the sequential transfer of the phosphoryl group from phosphoenolpyruvate (PEP) to the enzyme I (EI), histidine protein (HPr) and enzymes IIA (EIIA) and IIB (EIIB). The final step in this process involves the phosphorylation of the sugar imported by the transmembrane protein EIIC. .

The PTS was first described by the laboratory of Saul Roseman (Kundig et al. 1964) in 1964 in *E. coli* K235, as a novel system coupling sugar transport with sugar phosphorylation from phosphoenolpyruvate as the phosphoryl donor. Since then, PTSs have been identified in various species of bacteria as well as in archaea, transporting a wide range of monosaccharides, glycosides and polyols (Saier 2015). The PTS thus represents a major carbohydrate active-transport system in bacteria (Saier 2000). It is a multicomponent system involving both membrane-associated and cytoplasmic enzymes (Deutscher et al. 2006). The membranous component of the PTS system, enzyme II (EII), is made up of three to four subunits: IIA, IIB, IIC, and sometimes IID, which are specific either for a single substrate or, in a few cases, for a small group of closely related carbohydrates (Erni 2013). The cytoplasmic components are enzyme I (EI) and histidine protein (HPr), which are not carbohydrate-specific. Together, they constitute a phosphorylation cascade, allowing the phosphoryl group to be successively transferred from phosphoenolpyruvate to EI, to the HPr, then to subunits A and B of EII, and finally to the carbohydrate imported by the subunit EIIC or by the EIICD complex (Fig. 5) (Deutscher et al. 2014).

### Diversity of sequences and structures

Based on sequence similarities within the C domain of the EII complex, carbohydrate PTSs have been classified into seven families in the TCDB (Saier 2006): glucose-glucoside (4.A.1, Glc), fructose-mannitol (4.A.2, Fru), lactose-*N,N*-diacetylchitobiose (4.A.3, Lac), glucitol (4.A.4, Gut), galactitol (4.A.5, Gat), mannose-fructose-sorbose (4.A.6, Man), and l-ascorbate (4.A.7, l-Asc). It is noteworthy that some families include members that are nearly as distant from one another as they are from members of other families (Jeckelmann and Erni 2019). PTS family names are not always consistent with the functions of their members. For example, cellobiose PTS transporters are found in both the Glc family (Francl et al. 2010) and the Lac family (Kowalczyk et al. 2008), while fructooligosaccharide PTS transporters occur in the Glc family (Wang et al. 2020). Based on our review of the carbohydrate PTSs in the TCDB and in the literature (Supplementary Table 3), glycoside PTS transporters are only listed in the Glc, Lac, and Man families.

Depending on the family, the EII complexes of glycoside PTSs are encoded by one or more genes. The Glc family contains members with either a single multidomain protein, including the A, B, and C domains, or with two distinct proteins, one corresponding to the A domain and the other the B and C domains. Some members of the Glc family lack their own IIA domain and instead use the IIA domain of another system from the same family (Tchieu et al. 2001). By contrast, members of the Lac family always possess multidomain EII complexes classified into two subfamilies: one (4.A.3.1) consisting of two polypeptide chains (EIIA and EIIBC), and another (4.A.3.2) including three individual polypeptide chains (EIIA, EIIB, and EIIC). The Man family is the only PTS family whose members possess an additional IID domain. The EIIA, EIIB, EIIC, and EIID domains of glycoside PTSs in the Man family can be present either as four individual proteins or as three individual proteins, with the A and B domains located within the same protein. The Glc, Lac, and Fru families belong to a phylogenetic PTS-GFL superfamily (Nguyen et al. 2006) because of the structural homogeneity of their IIC domains, which display a uniform topology of 10 transmembrane helices (Cao et al. 2011, McCoy et al. 2016). Man family members are evolutionarily distinct from the GFL superfamily (Chen et al. 2012). In this family, the IIC domains probably consist of six or seven transmembrane helices, and the second integral membrane IID domains probably contains six transmembrane helices, according to the HMMTOP prediction (Tusnády and Simon 2001). However, the only solved structure of a mannose PTS transporter (ManYZ) from the Man family shows nine transmembrane helices in both the IIC (ManY) and IID (ManZ) domains (Liu et al. 2019). The structural organization and, consequently, the classification of glycoside PTSs, have a complex evolutionary history due to numerous horizontal gene transfer events, duplications, and non-orthologous displacements (Zúñiga et al. 2005).

### Functional diversity

Only a few dozen glycoside-targeting PTSs have been functionally characterized using one or more of the methods listed above (Supplementary Table 3). Most are specific for α- or β-linked glucosides, such as maltose, sucrose, and cellobiose, or their derivatives, such as N,N'-diacetylchitobiose and salicin, as the GFL superfamily is by far the largest (Jeckelmann and Erni 2019). Recently, transcriptomic analyses have revealed that, in *Lactobacillus plantarum* strain WCFS1, PTS1 (Saulnier et al. 2007, Panwar and Kapoor 2020) of the Glc family and PTS23C (Panwar and Kapoor 2020) of the Lac family might be involved in galactomannose/galactomannobiose and mannobiose utilization, respectively, although the specificity of these two PTSs still needs to be further characterized. In addition, within the Man family, a few PTSs process β-galactosides, β-mannosides, hyaluronic acid, and fucosyl-α-1,3-*N*-acetylglucosamine. Glycoside PTSs are thus mainly specific for disaccharides, although some can transport longer oligosaccharides with a DP of up to four. Other PTS transporters listed in Supplementary Table 3 were shown to be involved in the utilization of hyaluronic acid, glucomannooligosaccharides or lichenan oligosaccharides through transcriptional analysis or deletion of one of the PTS-encoding genes, but this does not mean that these PTSs can transport glycosides with a DP greater than four.

### Taxonomical diversity

We examined the taxonomical diversity of all characterized bacterial PTS transporters targeting glycosides derived from the TCDB Glc, Lac, and Man families and from the literature (Table 3). These 56 PTS transporters are spread across three phyla and 11 species, but more than half originate from species of the Bacillota phylum. The PTSs targeting glucosides are distributed across three phyla (Bacillota, Pseudomonadota, and Actinomycetota), whereas the PTSs targeting mannosides and galactosides are only found in species belonging to the Bacillota phylum. The PTS content is consistent with the species’ potential for facultative anaerobic and anaerobic growth (Jeckelmann and Erni 2019), which correlates with the fact that the Bacillota phylum is one of the dominant phyla in the mammalian gut microbiota. Thirty percent of the glycoside PTSs from Bacillota belong to the *Lactobacillus* genus; species from this genus can utilize diverse glycosides found in the human gut, allowing them to survive in this ecosystem. This trait, together with the fact that many *Lactobacillus* species provide benefits to the host’s general health and wellness, makes them useful as probiotics (Francl et al. 2010). Nevertheless, PTSs are also present in some pathogenic species, such as *Klebsiella pneumoniae* NTUH-K2044, which causes nosocomial and opportunistic infections (Wu et al. 2012), and *Vibrio cholerae* O1, which causes a dehydrating diarrheal illness (Meiborn et al. 2004). PTSs might thus play a role in habitat colonization by these strains and, consequently, in bacterial infection.

### Mechanism of glycoside import by PTS transporters

As described above, the transport of glycosides by PTSs is accompanied by a cascade of phosphorylation events, in which the phosphate moiety is transferred from phosphoenolpyruvate to the imported glycoside by a set of cytoplasmic phosphocarrier proteins (EI, HPr, EIIA, and EIIB). EI, HPr, and IIA are all phosphorylated at a histidine residue. The IIB domains of the GFL superfamily are phosphorylated at a cysteine residue, whereas the IIB domains of the mannose family are phosphorylated at a histidine residue. The crystal structures of several EI, HPr, IIA, and IIB proteins have been solved, either alone or in complex with other PTS proteins, and have been described in previous reviews (Robillard and Broos 1999, Peterkofsky et al. 2001, Deutscher et al. 2006, Erni 2013).

**Figure 6:**
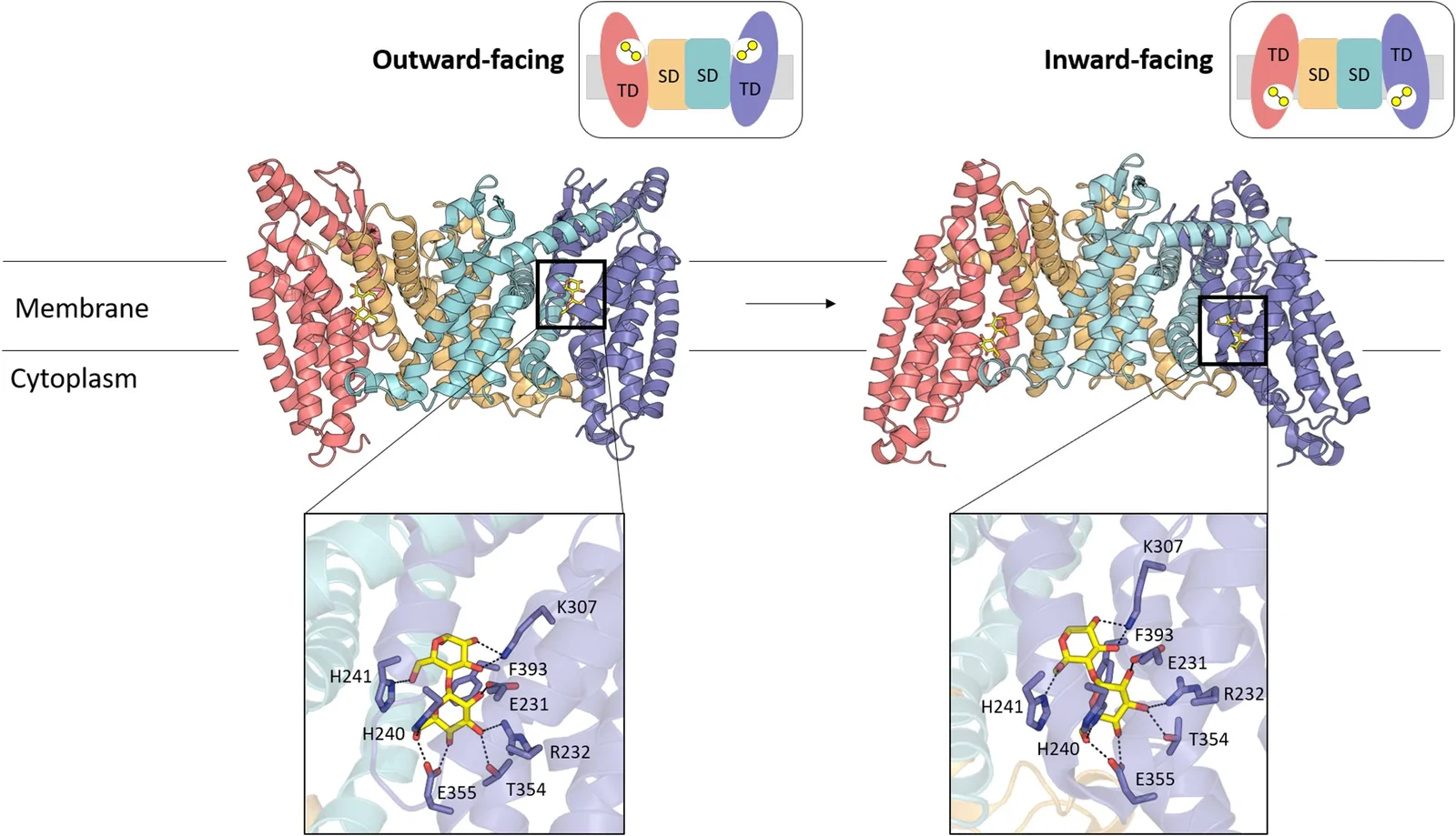
**Structure of the maltose-specific transporter EIIC from *Bacillus cereus* in outward-facing (PDB ID: 5IWS) and inward-facing (PDB ID: 6BVG) conformations**. The dimeric transporter contains a transport domain (TD) and a scaffold domain (SD) in each protomer. Maltose is transported across the membrane according to an elevator-type mechanism. Maltose is shown as sticks, with carbon atoms colored yellow and oxygen atoms colored red. Protein–maltose interactions are marked as dotted lines between the sugar and the residues involved.

Little was known about how the transmembrane EIIC domain selectively imports glycosides across the membrane until the high-resolution crystal structure of a diacetylchitobiose EIIC protein (bcChbC) from *Bacillus cereus* E33 L was solved in the inward-facing conformation (Cao et al. 2011). A few years later, the maltose-specific EIIC protein (bcMalT) from the same strain was solved in both inward-facing and outward-facing conformations (McCoy et al. 2016, Ren et al. 2018) (Fig. 6). Although bcChbC and bcMalT have different specificities, they belong to the same GFL superfamily and both proteins are homodimers containing 10 transmembrane helices that form a scaffold domain, containing the large and mostly hydrophobic protomer-protomer interface, and a transport domain containing the sugar-binding site and translocation pathway. An elevator mechanism was therefore proposed. Overall, the substrate first binds to the transporter (IIC) in the outward-facing open conformation; the substrate is then occluded and translocated across the membrane by rigid-body motion of the transport domain against the immobile scaffold domain, during which EIIC isomerizes to the inward-facing open conformation (Jeckelmann and Erni 2019). The isomerization rate is increased 100- to 1000-fold by the activity of the phosphorylated IIB domain and rendered unidirectional by concomitant phosphoryl transfer from the phosphorylated IIB domain to the substrate in the binding cavity and/or by the subsequent release of the phosphorylated substrate.

Recent cryo-EM analysis of the *E. coli* PTS glucose transporter captured in inward-, intermediate, and outward-facing conformations revealed protomer asymmetry in the transporter dimer structure, suggesting that each protomer functions independently (Roth et al. 2024, Roth and Fotiadis 2025). However, it is not known how IIB and IIC interact in the phosphate transfer process.

PTSs in members of the Man family contain a unique additional IID transmembrane domain. The only established structure of an EIICD complex is that of the *E. coli* K-12 protein specific for mannose (Liu et al. 2019), which also suggested a possible elevator mechanism. However, the feasibility of this mechanism for glycoside PTSs from the Man family still requires further investigation.

## MFS transporters of glycosides

**Figure 7:**
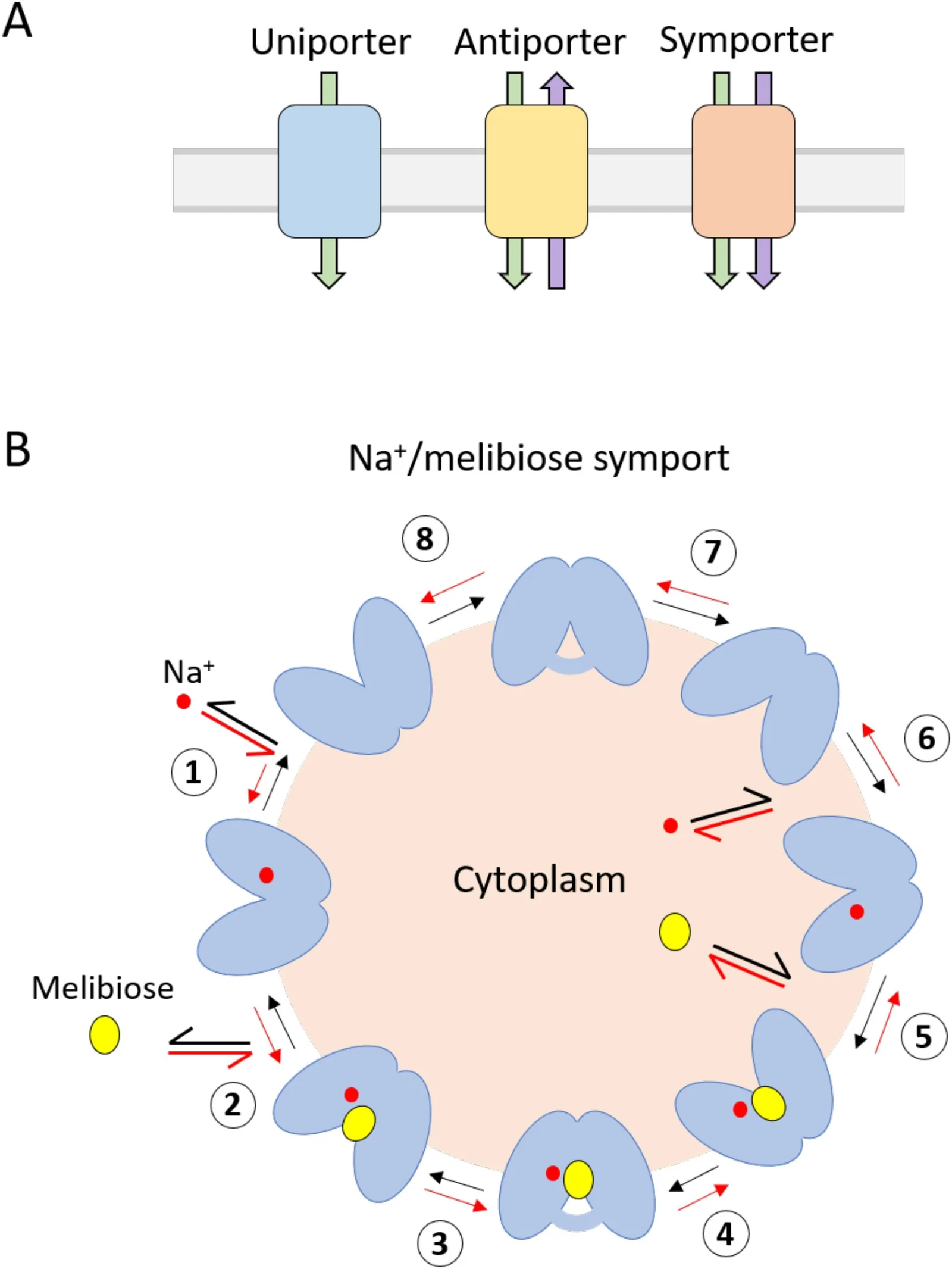
**Schematic representations of the MFS transport systems**. A) Schematic representation of the three types of MFS transporters. The uniporter catalyzes substrate diffusion across the membrane down its concentration gradient. The symporter and antiporter use the energy released from the downhill translocation of one substrate to drive the uphill translocation of another, either in the same (symporter) or opposite (antiporter) direction. B) Example of the Na^+^/melibiose symport, which is catalyzed by MelB from *Salmonella typhimurium*. Melibiose influx begins at step 1 and proceeds via the red arrows around the circle. Melibiose efflux transport begins at step 6 and proceeds via the black arrows around the circle. In both cases, only one melibiose molecule and one cation are transported across the membrane per cycle. Adapted from Guan and Hariharan (2021). .

Along with the ABC superfamily, the MFS is one of the two exceptionally large transporter superfamilies, with millions of sequenced members found ubiquitously in bacteria, archaea, and eukaryotes (Saier 2006). MFS transporters are single-polypeptide secondary carriers capable only of transporting small solutes in response to chemiosmotic ion gradients. The MFS superfamily constitutes the largest family of secondary membrane transporters. Its members can be classified as symporters, antiporters and, in the absence of a driving substance, uniporters (Fig. 7A) (Yan 2015). In the TCDB (2025 release), the MFS superfamily, annotated as TC# 2.A.1, is subdivided into 91 families, 7 of which have not yet been characterized. Each family contains members specific to a different set of related compounds, including monosaccharides, oligosaccharides, polyols, drugs, neurotransmitters, Krebs cycle metabolites, phosphorylated glycolytic intermediates, amino acids, peptides, organic anions, and inorganic anions (Reddy et al. 2012).

### Diversity of sequences and structures

In the TCDB, bacterial MFS transporters of glycosides are found in: (i) the Oligosaccharide:H^+^ Symporter (OHS) family (2.A.1.5), (ii) the Fucose:H^+^ Symporter (FHS) family (2.A.1.7), and (iii) the Glycoside–Pentoside–Hexuronide (GPH):Cation Symporter family (2.A.2) (Supplementary Table 4). The GPH family is a distant family within the MFS superfamily, since transporters from the GPH family show only marginal sequence similarity to members of the other families. Position-Specific Iterated BLAST (PSI-BLAST) analysis suggested that these proteins all arose from a common ancestor (Saier 2000). Glycoside MFS transporters are generally about 400–550 amino acids in length, with the exception of Gram-positive bacterial lactose permeases (LacS), which are larger because of an additional EII-like domain at the C-terminus, usually found in PTS transporters. Despite their low sequence similarity, all these proteins share the same topology, with 12 transmembrane helices, in which the first 6 transmembrane helices exhibit significant sequence similarity to the last 6 (Saier 2000).

### Functional diversity

The characterized bacterial MFS transporters involved in the uptake of different glycosides are listed in Supplementary Table 4. Transporters from the OHS family have been reported to be specific for a set of galactosides (such as lactose, raffinose, and melibose), as well as sucrose and maltose. Among the members of the OHS family, the LacY permease involved in the uptake of lactose and other galactosides by *E. coli* K-12 was the first MFS member to be investigated (Büchel et al. 1980) and remains one of the best-characterized MFS transporters (Kaback and Guan 2019).

Only one member of the FHS family, the ScrT transporter specific for sucrose uptake from *Shewanella frigidimarina* NCIMB 400, has been functionally characterized as being specific for oligosaccharide transport (Rodionov et al. 2010); the other characterized transporters from this family are involved in the transport of monosaccharides or their derivatives. By contrast, members of the GPH family exhibit broad specificity toward glycosides, including galactosides, glucosides, xylosides, and fructooligosaccharides.

Some transporters, including members of the OHS family, such as LacY from *E. coli* K-12 (King and Wilson 1990), CscB from *E. coli* EC3132 (Peng et al. 2009), MelY from *Enterobacter cloacae* ATCC 13047 (Shinnick et al. 2003, Tavoulari and Frillingos 2008), and the only characterized member of the GPH family, LacS from *Lactobacillus acidophilus* NCFM (Andersen et al. 2011) are able to transport different glycosides that share structural traits. For example, the LacY protein can internalize lactose, melibiose, thio-β-methyl galactopyranoside (TMG), 4-nitrophenyl-β-d-galactopyranoside, and 4-nitrophenyl-α-d-galactopyranoside because LacY contains a sugar-binding site that selectively recognizes the galactosyl moiety (Kaback and Guan 2019).

From the list of substrates of the characterized glycoside MFS transporters, these transporters appear to be mainly specific for disaccharides or their derivatives. However, MFS transporters can also import longer glycosides. Using gene deletion and growth dynamics analysis, two transporters were shown to be able to internalize oligosaccharides up to a DP of 4: the MFS transporter from an uncultured *Bacteroides* strain (Tauzin et al. 2016b) and FosT from *E. coli* BEN2908 (Schouler et al. 2009), which internalize xylotetraose and fructotetraose, respectively. Other MFS transporters, such as TogT (Hugouvieux-Cotte-Pattat and Reverchon 2001) from *Erwinia chrysanthemi* 3937 and LacS (Lba1463) (Andersen et al. 2011) from *Lactobacillus acidophilus* NCFM, have been shown to be responsible for growth on a mixture of oligosaccharides, but the size of the oligosaccharide they are able to transport has not been characterized. It is thus possible that MFS transporters can transport glycosides with a DP greater than 4, although further experiments are required to validate this hypothesis.

### Taxonomical diversity

The 18 characterized bacterial MFS transporters of glycosides span three phyla (Pseudomonadota, Bacillota, and Bacteroidota) (Table 4). Most of them (57.9%) belong to the Pseudomonadota phylum and 47.4% of these belong to the order Enterobacterales, including the well-known LacY from *E. coli*. The identification of LacY might have facilitated the identification of other Enterobacterales MFS, because gene transfer or duplication occurs more frequently intra-phyla than inter-phyla (Soucy et al. 2015). However, although most of the MFS identified to date belong to this order, these results may be biased because the analyses are based on the well-known LacY sequences from Pseudomonadota, and the vast majority of sequences likely remain unknown.

**Table 4:** Phylum and order distribution of characterized glycoside-targeting MFS transporters in bacteria.

Phylum	Order	Number of characterized MFSs	Percentage of MFSs characterized	
			Order	Phylum
Pseudomonadota	Enterobacterales	8	38.10	47.62
	Alteromonadales	1	4.76	
	Caulobacterales	1	4.76	
Bacillota	Lactobacillales	5	23.81	23.81
Bacteroidota	Bacteroidales	1	4.76	4.76
Actinomycetota	Bifidobacteriales	5	23.81	23.81

Glycoside MFS transporters are present in diverse bacterial phyla from different habitats, including the human gut and food and soil microbiota, suggesting that MFS transporters play an important role in bacterial survival and adaptation to their ecological niches. The phytopathogen *Erwinia chrysanthemi* 3937 (Hugouvieux-Cotte-Pattat and Reverchon 2001) uses a MFS to transport pectin oligomers, components of plant cell walls, thereby causing soft rot in plants. The fructooligosaccharide MFS transporter from the pathogenic *E. coli* BEN2908 strain is important for colonization of the chicken intestine, indicating a potential risk associated with high levels of fructooligosaccharide prebiotic usage in food and feed (Schouler et al. 2009). Nevertheless, glycoside MFS transporters are also found in strains that are beneficial to human health, such as the probiotic strains *Streptococcus thermophilus* A147 (Poolman et al. 1989), *Lactobacillus acidophilus* NCFM (Andersen et al. 2011) and *Lactobacillus pentosus* NZ9000 (Heuberger et al. 2001).

### Mechanism of glycoside import by MFS transporters

Before any crystal structures were solved, mutational analyses of some MFS transporters, such as the LacY (King and Wilson 1990), RafB (Camp et al. 2007), and MelB (Granell et al. 2010) proteins, enabled the identification of amino acid residues that play an important role in substrate recognition and transport across the membrane.

**Figure 8:**
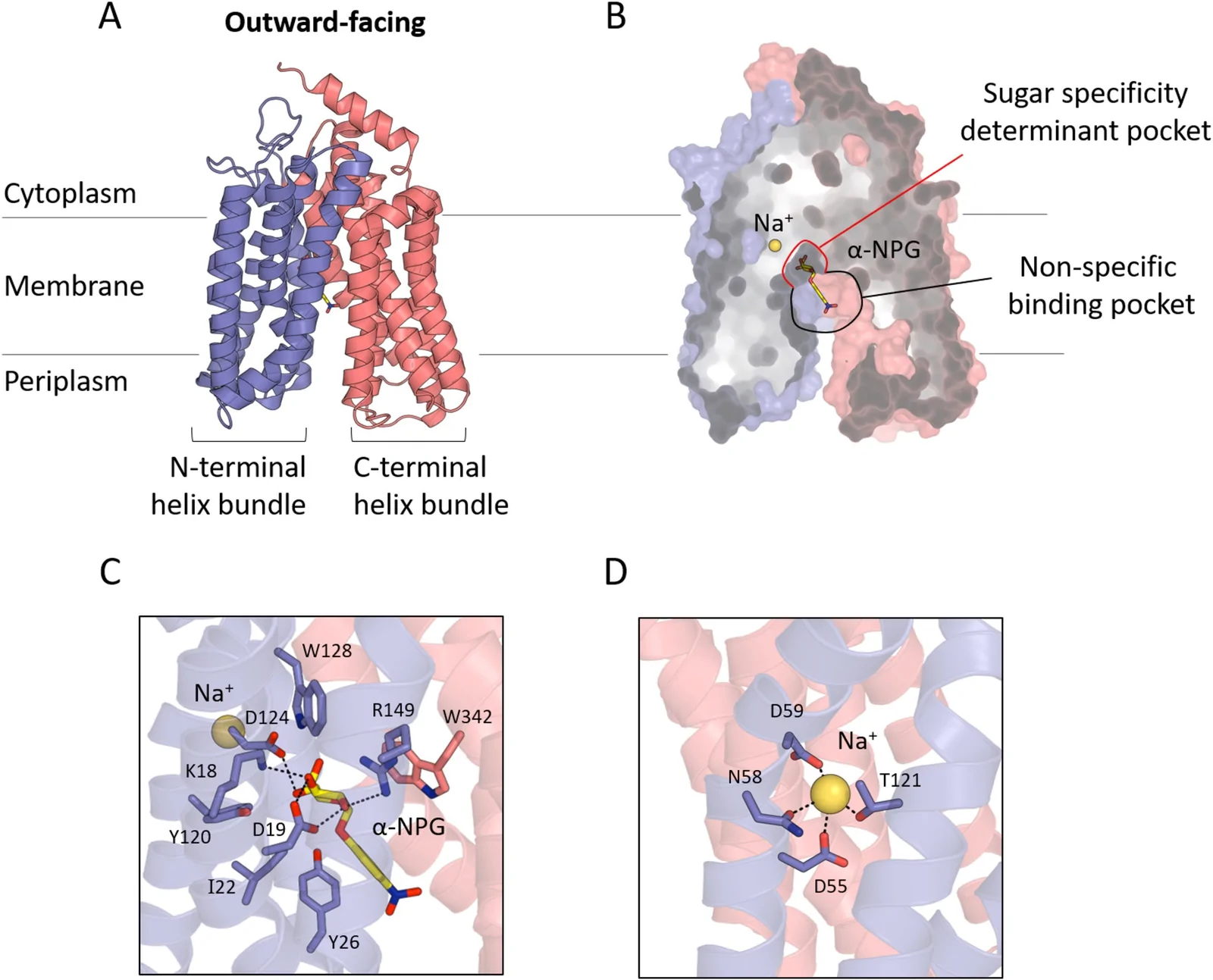
**The structure of the melibiose transporter MelB from *Salmonella typhimurium* is shown with its bound ligands, nitrophenyl-α-d-galactoside (α-NPG) and Na^+^**. A) Overall fold of the MelB transporter in an outward-facing conformation with bound α-NPG (PDB ID: 7L17). Both termini are located on the cytoplasmic side. B) The structure is shown in cross-section surface representation to highlight the ligands. The Na^+^ ion comes from the inward-facing Na^+^-bound cryoEM structure (PDB ID: 8T60), which was overlaid with the α-NPG-bound structure. The galactosyl moiety of α-NPG is located in a small pocket, forming multiple close contacts with the N-terminal helices and few with the C-terminal helices. C) Detailed interactions between α-NPG and residues from MelB in the outward-facing conformation (PDB ID: 7L17). Protein-ligand interactions are marked as dotted lines. D) Detailed interactions between Na^+^ and residues from MelB in the inward-facing conformation (PDB ID: 8T60). Na^+^ is represented as a yellow sphere. Protein-oligosaccharide interactions are marked as dotted lines between the sugar and residues.

The first glycoside MFS structure to be solved was that of the lactose permease (LacY) from *E. coli* K-12, in an inward-facing conformation with a bound lactose homolog, β-d-galactopyranosyl-1-thio-β-d-galactopyranoside (Abramson et al. 2003), and subsequently in an inward-open conformation (Guan et al. 2007). More recently, the structure of a second glycoside MFS transporter, the melibiose-specific MelB protein from *Salmonella typhimurium* LT2, was solved in an outward-facing state with a bound ligand, either nitrophenyl-α-d-galactoside or dodecyl-β-d-melibioside and an inward-facing state with Na^+^(Guan and Hariharan 2021, Hariharan et al. 2023) (Fig. 8). These structures enabled the general features of MFS transporters to be described (Fig. 7B). A canonical MFS fold comprises two domains, the N- and C-terminal domains, each consisting of six consecutive transmembrane helices. A single substrate-binding cavity is located at the center of the membrane and enclosed by the N- and C-terminal domains. Moreover, an alternating-access mechanism has been proposed to explain the translocation process (Zhang et al. 2015, Kaback and Guan 2019, Sauve et al. 2023). Basically, the transporters adopt three states; (i) the inward-facing state where the binding site is on the cytoplasmic side, (ii) the occluded state where the transporter is not open to either side of the membrane as a result of the helices that surround the substrate being compact, thus the substrate is unable to enter or leave the binding site, and (iii) the outward-facing state where the binding space is on the extracellular side. They alternate between these conformations to translocate substrates across the membrane.

## Concluding remarks

Glycoside transporters play an essential role in bacterial metabolism, enabling the import of various glycosidic substrates required for bacterial survival and growth in diverse environments. This review focused on four transporter superfamilies, namely the TonB-dependent transporters (TBDT), the ABC system, the PTS, and the MFS, highlighting their taxonomical, functional, mechanistic, and structural diversity in bacteria.

Over the last decade, the development of culturing and metagenomic approaches has enabled access to an exponentially increasing number of transporter sequences from both cultured and uncultured bacteria. However, the vast majority of these transporter sequences remain uncharacterized.

Methodological opportunities have developed to refine the characterization of uptake specificity by discriminating it from binding specificity.

- Droplet microfluidics approaches encapsulating single cells enable profiling of transport specificity toward structurally defined oligosaccharides, which can be very expensive commercially. This approach increases screening throughput and reduces screening costs by at least three orders of magnitude.
- The use of recombinant hosts, for which genetic tools are available to modify metabolism and enable co-production of several recombinant proteins (the different proteins constituting certain transporters, but also the CAZymes encoded in the same loci), appears very promising when it comes to simplifying these systems and avoiding the functional redundancy observed in native strains. The use of recombinant hosts is also essential for studying transporters from uncultured bacteria. Recently, *E. coli* was shown to be a useful host for expressing genes from PULs cloned into fosmids, and for producing functional transporters from uncultured species. Nevertheless, control of the concomitant expression of each element of the locus is crucial.

The lack of functional data limits the understanding of transporter structure-function relationships in general. It is also a major barrier to predicting their precise substrate targets. Sequence similarity networks (SSN) enable rapid clustering of sequences and thus reveal experimentally unexplored sequences that may exhibit novel functionalities (Copp et al. 2018, Wang et al. 2020). Once experimental data are sufficiently enriched, SSNs could also be used to divide the large, multifunctional transporter families into more specific subfamilies, as is currently being done for CAZyme families (Hornung and Terrapon 2023, Lebreton et al. 2025).

Until recently, X-ray crystallography was the only method available for determining the 3D structure of transporters. In the last decade, the emergence of modern cryo-EM has opened up new possibilities in this field, as it enables the structure determination of transporters that do not crystallize or do not diffract. It also enables the simultaneous study of these multiprotein complexes and their conformational dynamics.

In the future, the study of transporter structure-function relationships will benefit greatly from artificial intelligence (AI), particularly in molecular docking and thermodynamics-based molecular dynamics used to analyze ligand binding and channeling through transmembrane domains. AI will also fuel structure prediction (e.g. AlphaFold-Multimer) and the capture of alternative conformations.

From a biotechnological perspective, an upcoming challenge is the de novo design of glycoside transporters for the uptake of desired molecules in chassis strains to enhance fermentation processes. From a public health viewpoint, a better understanding of the relationships between structure, specificity and efficiency could lead to improved control over the functioning and composition of the human microbiota. This could be achieved by using oligosaccharides as nutraceuticals that can be utilized specifically by beneficial species, or by developing specific transporter inhibitors as drugs to prevent pathogen growth. Overall, glycoside transporters represent a dynamic field of research with significant implications for biotechnology and medicine.

## Supplementary Material

fuag040_Supplemental_File
