# Supplementary material for: Functional, structural, and taxonomical diversity of glycoside transporters in bacteria
Source: FEMS Microbiol Rev. 2026 Sep 2;50:fuag040. doi: 10.1093/femsre/fuag040 (PMC13543919; doi:10.1093/femsre/fuag040)
Supplement: Supplementary file 1 [file fuag040_supplemental_file.docx]

**Supplementary Table 1. Summary of the SusD and SusC/D pairs involved in polysaccharide utilization by Bacteroidota, characterized either biochemically, or by gene deletion or mutation.**

Unless they were also characterized by one of these techniques, this list does not include proteins whose genes have been shown to be upregulated during polysaccharide utilization by transcriptomic analysis (listed in the PULDB).

| **Protein name**  **(locus tag)** | **Substrate** | | **Organism** | **Characterization method** | | **Structure** | | | **Reference** | |
| --- | --- | --- | --- | --- | --- | --- | --- | --- | --- | --- |
| **SusD proteins** | | | | | | | | | | |
| BT3701 | Starch Maltooligosaccharide | *B. thetaiotaomicron* | | ITC  Gene deletion | | 3CKC apo  3CKB maltotriose  3CK9 maltoheptaose  3CK8 β-cyclodextrin  3CK7 α-cyclodextrin | | | (Koropatkin *et al.* 2008) | |
| BT1043 | *N*-acetyllactosamine | *B. thetaiotaomicron* | | ITC | | 3EHM apo  3EHN *N*-acetyllactosamine | | | (Koropatkin *et al.* 2009) | |
| SusD1 (JQ755420)  SusD2  (JQ755421) | Cellulose | Uncultured Svalbard reindeer rumen bacterium assigned to Bacteroidota | | Pull down assay  Indirect immunofluorescence microscopy | | - | | | (Mackenzie *et al.* 2012) | |
| BACOVA_02651  SGBP-A | Xyloglucan | *B. ovatus* | | AGE  ITC  Gene deletion  Point mutation | | 5E75 apo  5E76 xylo-glucooligosaccharide | | | (Larsbrink *et al.* 2014; Tauzin *et al.* 2016a; Foley *et al.* 2019) | |
| BT3984 | Mannan | *B. thetaiotaomicron* | | ITC  Gene deletion | | - | | | (Cuskin *et al.* 2015) | |
| BACOVA_04392 | Birchwood glucuronoxylan  Wheat arabinoxylan | *B. ovatus* | | ITC | | - | | | (Rogowski *et al.* 2015) | |
| BACOVA_03427 | Birchwood glucuronoxylan  Corn glucuronoarabinoxylan |  |  |  |  |  |  |  |  |  |
| Fjoh_4558 | Chitin | *F. johnsoniae* ATCC 17061 | | SDS-PAGE  ITC  Gene deletion | | 5J90 apo  5J5U apo | | | (Larsbrink *et al.* 2016) | |
| Fjoh_4561 |  |  |  |  |  |  |  |  |  |  |
| F5_SusD | Xylooligosaccharides | Uncultured *Bacteroides* | | Gene deletion  Growth assay  HPAEC-PAD  SDS-PAGE  AGE  ITC  DSC | | 7NEK apo | | | (Tauzin *et al.* 2016b, 2022) | |
| BACOVA_02095 | Galactomannan  Glucomannan | *B. ovatus* | | AGE  Gene deletion | | - | | | (BAGENHOLM *et al.* 2017) | |
| BT3311 | 1,6-β-glucan | *B. thetaiotaomicron* | | AGE  ITC | | - | | | (Temple *et al.* 2017) | |
| BT4659 | Heparin/heparan sulfate | *B. thetaiotaomicron* | | ITC | | 3IHV apo | | | (Cartmell *et al.* 2017) | |
| SAMN04488552_1347 | Laminarin | *Gramella*  sp. MAR_2010_102 | | AGE  Point mutation | | 6GCZ apo | | | (Mystkowska *et al.* 2018) | |
| BT4113 | Homogalacturonan | *B. thetaiotaomicron,* | | Gene deletion  ITC | | - | | | (Luis *et al.* 2018) | |
| BT4670 | Pectic galactan |  |  |  |  |  |  |  |  |  |
| BoManPUL SusD-like | Galactomannan, glucomannan | *B. ovatus* | | MST | | - | | | (Bågenholm *et al.* 2019) | |
| BACOVA_02743 | β(1,3)/β(1,4)-glucans | *B. ovatus* | | AGE  ITC  Gene deletion  Point mutation | | 6E60 apo  6DMF cellohexaose  6E61 heptasaccharide | | | (Tamura *et al.* 2019) | |
| BT3331 | Glycosaminoglycan chondroitin sulfate | *B. thetaiotaomicron* | | ITC | | - | | | (Ndeh *et al.* 2020) | |
| BuSGBP-A (BACUNI_01488) | No binding | *B. uniformis* | | AGE  ITC  Point mutation | | 7KV1 apo | | | (Déjean *et al.* 2020; Tamura *et al.* 2021) | |
| BtSGBP-A | β(1,3)-glucan | *B. thetaiotaomicron* | |  |  | 7KV2 apo  7KV3 laminarihexaose | | |  |  |
| BfSGBP-A (HMPREF9446_00614) | β(1,3)-glucan | *B. fluxus* | |  |  | 7KV4 apo | | |  |  |
| BuXyGUL1 SGBP-A  BuXyGUL2 SGBP-A | Xyloglucan oligosaccharides Xyloglucan and xyloglucan oligosaccharides | *B. uniformis ATCC 8492* | | ITC | | - | | | (Grondin *et al.* 2022) | |
| FCWusD (Cpin_2192) | Curdlan,  yeast β-glucan, lichenan, and barley β-glucan | *Chitinophaga pinensis* | | Pull-down assays | | - | | | (Lu *et al.* 2023) | |
| AqSusD | Chitin and chitin oligosaccharides | *Aquimarina sp. SCSIO 21287* | | Pull-down assay  ITC Point mutations | | 8J0P apo  8JXZ triacetyl-β-chitotriose | | | (Yang *et al.* 2023) | |
| SusD (P162_RS13765) | Glycogen, pullulan | *Flavimarina sp. Hel_I_48* | | AGE | | | - | | | (Beidler *et al.* 2023) |
| SusD_I_2 (P162_RS02355) | Beechwood xylan, P*almaria palmata* xylan, rye arabinoxylan, wheat arabinoxylan of medium viscosity. | *Flavimarina sp. Hel_I_48*  Comparative genomics | | | Proteome analysis  AGE | | | - | | (Dutschei *et al.* 2023) |
| BT3089 | Dextran | *B. thetaiotaomicron* | | Gene deletion  Binding tracking by fluorescent polymers  Cryo-EM | | - | | | (White *et al.* 2022; Wong *et al.* 2024) | |
| FjDusD (Fjoh_4433) | Isomaltooligosaccharides | *F. johnsoniae* | | AGE  ITC  AlphaFold | | - | | | (Nakamura *et al.* 2023) | |
| 41O1_SusD | Laminaritriose, laminarins, yeast-β-glucan | Uncultured *Bacteroides* from the cow rumen | | AGE  Thermal shift analysis  NMR spectroscopy AlphaFold | | - | | | (Li *et al.* 2024) | |
| ScSGBP-A (Prevcop_05098) | β(1,3)/β(1,4)-glucan | *S. copri* DSM 18205 | | AGE  ITC | | 9BAM apo  9BAL cellopentose | | | (Golisch *et al.* 2024) | |
| **SusC/D complexes** | | | | | | | | | | |
| BT2263-2264 | In complex with 10-residue peptide(s) | | *B. thetaiotaomicron* | X-ray crystallography | | 5FQ6 | | | (Sonnenburg *et al.* 2010; Glenwright *et al.* 2017) | |
| BT1762-1763 | In complex with levan fructo-oligosaccharides | | *B. thetaiotaomicron* | X-ray crystallography  Cryo-EM | | 5T3R apo  6Z9A oligo DP7  6ZAZ oligos DP4, DP7  8A9Y apo utilisome  8AA0 utilisome with DP4-7  8AA1 oligos DP4-7  8AA2 utilisome with oligos DP4-7-13  8AA3 oligos DP4-7 | | | (White et al. 2023 ; Gray et al. 2021 ; Glenwright et al. 2017) | |
| BT3089-3090 | Dextran (apo-structure) | | *B. thetaiotaomicron* | Cryo-EM | | 8AA4 | | | White et al. 2023 | |

**Supplementary Table 2. List of the bacterial ABC transporters involved in glycoside utilization, characterized either by binding or transport assays, gene deletion and/or transcriptional approaches.**

The glycoside ABC transporters retrieved from the literature but which are not listed in the TCDB were classified based on their conserved domains by using a BLAST analysis against the NCBI’s Conserved Domain Database (Marchler-Bauer *et al.* 2017; Panwar, Shubhashini and Kapoor 2023).)

| **TCDB Family name** | **TCID** | **Protein name**  **(locus tag of SBP)** | **Substrate** | **Organism** | **Characterization method** | **Reference** |
| --- | --- | --- | --- | --- | --- | --- |
| CUT1 | 3.A.1.1.1 | MalEFGK  (b4034) | Maltooligosaccharides (DP2-7) | *E. coli* K-12 | Gene deletion  Binding test  Transport assay  Crystal structure | (Wandersman, Schwartz and Ferenci 1979; Oldham *et al.* 2007; Khare *et al.* 2009; Oldham and Chen 2011; Oldham, Chen and Chen 2013) |
|  | 3.A.1.1.2 | YufKLM  (BSU34160) | Galactooligosaccharides (DP2-4) | *B. subtilis* KM0 | Binding test | (Watzlawick, Heravi and Altenbuchner 2016) |
|  | 3.A.1.1.9 | GuoEFGK | Xylooligosaccharides (DP2-6) | *G. stearothermophilus* T-6 | Binding test | (Shulami *et al.* 1999, 2007) |
|  | 3.A.1.1.10 | AlgSM1M2Q1Q2 | Alginate (DP 2-7) | *Sphingomonas* *sp*. strain A1 | Transport assay  Gene deletion  Crystal structure | (Momma *et al.* 2000; Mishima *et al.* 2003; Maruyama *et al.* 2015; Kaneko *et al.* 2017)  (Murata, Kawai and Hashimoto 2022) |
|  | 3.A.1.1.11 | TogABMN | Oligogalacturonides (DP2-3) | *E. chrysanthemi* 3937 | Gene deletion  Heterologous expression in *E. coli* | (Hugouvieux-Cotte-Pattat and Reverchon 2001; Hugouvieux-Cotte-Pattat, Blot and Reverchon 2001) |
|  | 3.A.1.1.17 | ThuEFGK  (CN159_00480) | Trehalose, maltose, sucrose | *S. meliloti* Rm1201 | Gene deletion  Transport assay | (Jensen, Peters and Bhuvaneswari 2002) |
|  | 3.A.1.1.18 | NacEFG | *N*-Acetylglucosamine, chitobiose | *S. olivaceoviridis* | Transport assay  Binding test  Gene deletion | (Xiao *et al.* 2002) |
|  | 3.A.1.1.19 | PalEFGK | Platinose (Isomaltulose) | *A. tumefaciens* MAFF301001 | Gene deletion | (De Costa, Suzuki and Yoshida 2003) |
|  | 3.A.1.1.20 | MsmEFGK  (D7H66_05730) | Fructooligosaccharides (DP3-5) | *L. acidophilus* NCFM | Transcriptional analysis  Gene deletion | (Barrangou *et al.* 2003) |
|  | 3.A.1.1.21 | BxlEFGK  (SCO7028) | Xylobiose, xylotriose | *S. thermoviolaceus* OPC-520 | Transcriptional analysis  Binding test | (Tsujibo *et al.* 2004) |
|  | 3.A.1.1.22 | MalE1E2FGK  (TM_1204) | Maltose, maltotriose, mannotetraose (MalE1) | *T. maritima* MSB8 | Transcriptional analysis  Binding test | (Nanavati, Nguyen and Noll 2005) |
|  | 3.A.1.1.22 | MalE1E2FGK  (TM_1839) | Maltose, maltotriose, trehalose (MalE2) | *T. maritima* MSB8 | Transcriptional analysis  Binding test | (Nanavati, Nguyen and Noll 2005) |
|  | 3.A.1.1.23 | CebEFG | Cellobiose, cellotriose | *S. reticuli* DSM 40776 | Transcriptional analysis  Gene deletion  Transport assay | (Schlösser *et al.* 1999) |
|  | 3.A.1.1.25 | -  (TTC1627) | Trehalose, maltose, sucrose, palatinose | *T. thermophilus* HB27 | Transport assay  Binding test  Crystal structure | (Silva *et al.* 2005; Chandravanshi *et al.* 2019b) |
|  | 3.A.1.1.26 | MalEFG  (BSU34610) | Maltodextrin | *B. subtilis* 168 | Transport assay  Binding test  Gene deletion | (Schönert *et al.* 2006) |
|  | 3.A.1.1.27 | MalXFGK  (SMU_1568) | Maltodextrin (DP2-7) | *S. mutans* ATCC 700610 | Transport assay  Gene deletion | (Webb, Homer and Hosie 2008) |
|  | 3.A.1.1.28 | MsmEFGK  (SMU_878) | Raffinose, stachyose | *S. mutans* ATCC 700610 | Gene deletion | (Webb, Homer and Hosie 2008) |
|  | 3.A.1.1.31 | LpqY-SugA-SugB-SugC | Trehalose | *M. tuberculosis* H37Rv | Transport assay  Gene deletion | (Kalscheuer *et al.* 2010)  (Cassio Barreto de Oliveira and Balan 2020; Liu *et al.* 2020; Sabharwal *et al.* 2020; De la Torre *et al.* 2021; Furze *et al.* 2021; Sharma *et al.* 2022) |
|  | 3.A.1.1.32 | GgtABCD  (slr0529) | Sucrose, trehalose | *Synechocystis* *sp*. strain PCC6803 | Gene deletion  Binding test | (Mikkat and Hagemann 2000) |
|  | 3.A.1.1.33 | DasABC | Chitobiose | *S. coelicolor* A3(2) | Transcriptional analysis  Gene deletion | (Saito *et al.* 2008) |
|  | 3.A.1.1.34 | AraNPQ | α-1,5-Arabinooligosaccharides (DP3-4) | *B. subtilis* IQB496 | Gene deletion | (Ferreira and De Sá-Nogueira 2010) |
|  | 3.A.1.1.41 | TreEGF | Trehalose | *T. maritima* MSB8 | Binding test | (Boucher and Noll 2011) |
|  | 3.A.1.1.44 | MalEFG  (SCO2231) | Maltose | *S. coelicolor* A3(2) | Gene deletion | (Van Wezel *et al.* 1997) |
|  | 3.A.1.1.45 | MusEFGK  (Cgl2461) | Maltose | *C. glutamicum* ATCC 13032 | Transcriptional analysis  Gene deletion | (Henrich *et al.* 2013) |
|  | 3.A.1.1.48 | MalEFGK  (BLJ_1686) | Lacto-*N*-biose,  galacto-*N*-biose | *B. longum* JCM1217 | Binding test | (Suzuki *et al.* 2008) |
|  | 3.A.1.1.51 | MalEFGK  (Bd1227) | Maltooligosaccharides (DP2-7) | *B. bacteriovorus* ATCC 15356 | Gene deletion  Binding test  Transport assay  Crystal structure | (Licht *et al.* 2019) |
|  | 3.A.1.1.52 | GlsQPCD  (DSM107007_RS23065) | Sucrose, maltose, esculin | *Anabaena* sp. PCC 7120 | Gene deletion | (Nieves-Morión and Flores 2018) |
|  | 3.A.1.1.53 | RafEFGK  (BIF_01592) | α-1,6-Glucosides and galactosides (2-7) | *B. animalis* subsp. *lactis* Bl-04 | Transcriptional analysis  Binding test  Crystal structure | (Ejby *et al.* 2016) |
|  | 3.A.1.1.60 | UspABC | Peptidoglycan precursors | *M. tuberculosis (Mtb)*  *M. smegmatis mc2155* | Gene deletion  Operon analysis  Topology mapping  Proteomic analysis  Phylogenetic analysis  Crystal structure | (Cassio Barreto de Oliveira and Balan 2020; De la Torre *et al.* 2021; Karlikowska *et al.* 2021) |
|  | 3.A.1.1.61 | Rv2038c-0Rv2041c | Sugar | *M. tuberculosis* H37Rv | Phylogenetic analysis  Crystal structure | (Cassio Barreto de Oliveira and Balan 2020; De la Torre *et al.* 2021) |
|  | Not described | XynEFG | Xylooligosaccharides (DP2-6) | *G. stearothermophilusi* T-6 | Transcriptional analysis  Binding test | (Shulami *et al.* 2007) |
|  | Not described | CbpA | Cellotriose | *C. thermocellum* DSM 1237 | Binding test | (Nataf *et al.* 2009) |
|  | Not described | CbpB | Cellooligosaccharides (DP2-5) | *C. thermocellum* DSM 1237 | Binding test | (Nataf *et al.* 2009) |
|  | Not described | CbpC | Cellooligosaccharides (DP2-5) | *C. thermocellum* DSM 1237 | Binding test | (Nataf *et al.* 2009) |
|  | Not described | CbpD | Cellooligosaccharides (DP3-5) | *C. thermocellum* DSM 1237 | Binding test | (Nataf *et al.* 2009) |
|  | Not described | LBP | Laminaribiose | *C. thermocellum* DSM 1237 | Binding test | (Nataf *et al.* 2009) |
|  | Not described | GacHFG | Acarbose, maltooligosaccharides (DP2-4) | *S. glaucescens* GLA.O | Binding test  Crystal structure | (Vahedi-Faridi *et al.* 2010) |
|  | Not described | AbnEFG | Arabinooligosaccharides (DP3-8) | *G. stearothermophilusi* T-6 | Binding test | (Shulami *et al.* 2011) |
|  | Not described | XBP1 | Xyooligosaccharides (DP2-4) | *C. polysaccharolyticus* ATCC BAA-17 | Binding test  Crystal structure | (Han *et al.* 2012) |
|  | Not described | BlAXBP  (Balac_0514) | Xylooligosaccharides (DP2-6), arabinoxylan-oligosaccharides (DP3-5) | *B. animalis* subsp. *lactis* Bl-04 | Binding test  Crystal structure | (Ejby *et al.* 2013) |
|  | Not described | CpMnBP1 | (Galacto-) Mannooligosaccharides (DP2-5) | *C. polysaccharolyticus* ATCC BAA-17 | Binding test  Crystal structure | (Chekan *et al.* 2014) |
|  | Not described | -  (EUR_01830) | Mannooligosaccharides (DP3-7), acarbose | *E. rectale* DSM 17629 | Proteomic analysis  Binding test | (Cockburn *et al.* 2014) |
|  | Not described | -  (EUR_31480) | Mannooligosaccharides (DP2-3) | *E. rectale* DSM 17629 | Proteomic analysis  Binding test | (Cockburn *et al.* 2014) |
|  | Not described | -  (EUR_21010) | Cellooligosaccharides (DP2, 3) | *E. rectale* DSM 17629 | Proteomic analysis  Binding test | (Cockburn *et al.* 2014) |
|  | Not described | CsnEFG | Chitosan-derived oligosaccharides; Glucosamine oligosaccharides (GlcN)_n_ | *S. coelicolor A3(2)* | Gene deletion  Transport assay | (Viens *et al.* 2015) |
|  | Not described | xylEFG ABC transporter | Xyloside, Arabino-xylooligosaccharides, xylooligosaccharides | *C. glutamicum* | Gene deletion | (Watanabe *et al.* 2015) |
|  | Not described | GanSPQ-MsmX | Galacto-oligosaccharides (DP3, DP4), galactotetraose, galactotriose  Cyclodextrin | *B. subtilis* | Surface plasmon resonance (SPR) spectroscopy  Fluorescence and circular dichroism (CD) spectroscopy  Gene deletion  TSA | (Watzlawick et al 2016; Kamionka and Dahl 2001) |
|  | Not described | SBP_2458_  (Ccel_2458) | Xyloglucan dextrins | *R. cellulolyticum* | Transcriptional analysis Binding test  Gene interruption | (Ravachol *et al.* 2016) |
|  | Not described | ManHIJ | (Galacto-) Mannooligosaccharides (DP3-5) | *Bacillus* sp. N16-5 | Transcriptional analysis  Binding test  Gene deletion | (Song *et al.* 2016) |
|  | Not described | FL-SBP | Fucosyllactose | *B. breve* BR-A29 | Gene deletion | (Matsuki *et al.* 2016) |
|  | Not described | FusABC  (SP_1796) | Fructooligosaccharides (DP3-5) | *S. pneumoniae* TIGR4 | Transcriptional analysis  Binding test  Gene deletion  Crystal structure | (Linke *et al.* 2013; Culurgioni *et al.* 2017) |
|  | Not described | SfuABC  (SPV_1585) | Fructooligosaccharides (DP3-5) | *S. pneumoniae* D39 | Binding test  Gene deletion  Crystal structure | (Culurgioni *et al.* 2017) |
|  | Not described | CpSBC4535 | α-1,5- Arabinooligosaccharides (DP 3-6) | *C. polysaccharolyticus*  ATCC BAA-17 | Transcriptional analysis  Binding test  Gene deletion | (Wefers *et al.* 2017) |
|  | Not described | CuaABC  (Ccel_2112) | Cellooligosaccharides (DP2-5) | *R.*  *cellulolyticum*  H10 ATCC 35319 | Binding test | (Fosses *et al.* 2017) |
|  | Not described | -  (Smon_0123) | Sulfated GalNAc | *S. moniliformis* DSM 12112 | Binding test  Crystal structure | (Oiki *et al.* 2017) |
|  | Not described | MdxEFG  (HMPREF9496_01133) | Maltooligosaccharides (DP4-7) | *E. faecalis* JH2-2 | Gene deletion | (Sauvageot *et al.* 2017) |
|  | Not described | CycB-GanPQ (GanSPQ) | Galactan; Nonlinear arabinooligosaccharides; Galactooligosaccharides | *B. subtilis* | Transcriptional analysis  Gene deletion | (Ferreira, Mendes and de Sá-Nogueira 2017) |
|  |  | YtcQP-YteP | Polygalacturonan and rhamnogalacturonan |  |  |  |
|  |  | YesOPQ | Polygalacturonan and rhamnogalacturonan |  |  |  |
|  | Not described | PglK | Lipid-linked-oligosaccharide (LLO), GlcGalNAc5Bac-PP-undecaprenyl | *C. jejuni* | Crystal structure  *In vitro* tLLO flipping assay  *In vivo* LLO flipping assay  Mass Spectrometry (MS) | (Perez *et al.* 2015, 2017, 2019) |
|  | Not described | RiXBP  (ROSINTL182_08199) | Xylooligosaccharides (DP3-6), arabinoxylotriose, arabinoxylotetraose | *R. intestinalis* L1-82 | Transcriptional analysis  Binding test | (Leth *et al.* 2018) |
|  | Not described | Wzm–Wzt | O-antigen polysaccharide | *A. aeolicus* | Crystal structure | (Bi *et al.* 2018; Jones and George 2020) |
|  | Not described | RiMnBP  (ROSINTL182_05479) | β-1,4-Mannooligosaccharides (DP3-5) | *R. intestinalis* L1-82 | Binding test | (La Rosa *et al.* 2019) |
|  | Not described | BlMnBP1  (BLAC_00780) | β-1,4-Mannooligosaccharides (DP3-6) | *B. animalis* subsp. *lactis* ATCC27673 | Transcriptional analysis  Binding test  Crystal structure | (Ejby *et al.* 2019) |
|  | Not described | BlMnBP2 (BLAC_00785) | β-1,4-Mannooligosaccharides (DP3-6) | *B. animalis* subsp. *lactis* ATCC27673 | Transcriptional analysis  Binding test  Crystal structure | (Ejby *et al.* 2019) |
|  | Not described | Bal6GBP  (Balac_0483) | β-(1,3/6)-Galactosides (DP2, 4) | *B. animalis* subsp. *Lactis* Bl-04 | Binding test  Crystal structure | (Theilmann *et al.* 2019) |
|  | Not described | TTHA0356 | Trehalose, maltose | *T. thermophilus HB8* | Crystal structure | (Chandravanshi *et al.* 2019a) |
|  |  | TTHA1652 | Cyclodextrin, maltodextrin (4–5 pyranose units) |  |  |  |
|  |  | TTHB082 | β-glucosides (laminaribiose, cellobiose) |  |  |  |
|  | Not described | cphy2464-cphy2466 | Cellobiose | *C. phytofermentans ISDg (ATCC 700394)* | Gene deletion  Binding test | (Cerisy *et al.* 2019) |
|  | Not described | MsmEX-AmyCD | Melibiose, raffinose and stachyose | *B. subtilis* | Transcriptional analysis  Gene deletion  Binding test | (Morabbi Heravi, Watzlawick and Altenbuchner 2019) |
|  | Not described | GltABC | Lacto-*N*-biose I/galacto-*N*-biose, lacto-*N*-tetraose (LNT) | *B. longum 105-A* | Crystal structure  Gene deletion  Transcriptional analysis  Binding test | (Sakanaka *et al.* 2019) |
|  |  | FL1-BP (Blon_0341–0343) | Fucosyllactose (FL)  2’-FL, 3-FL | *B. infantis JCM 1222^T^* |  |  |
|  |  | FL2-BP (Blon_2202–2204) | Fucosyllactose (FL)  2’-FL, 3-FL, lactodifucotetraose (LDFT) and lacto-*N*-fucopentaose (LNFP) I | *B. infantis JCM 1222^T^* |  |  |
|  | Not described | GalCDE | Galactooligosaccharide (GOS) | *Bifidobacteria :*  *B. breve UCC2003*  *B. infantis strain DSM 20088*  *B. breve strain DSM 20091*  *B. thetaiotaomicron* | Transport assay  Transcriptional analysis | (Böger *et al.* 2019) |
|  |  | GosDEC | Galactooligosaccharide (GOS) | *Bifidobacteria :*  *B. lactis Bl-04*  *B. lactis strains W51–W53*  *B. breve strain DSM 20091*  *B. infantis strain DSM 20088* |  |  |
|  | Not described | BABP (BLLJ_0208) | β-l-arabinobiose | *B. longum* | Binding test  Crystal structure | (Miyake *et al.* 2019) |
|  | Not described | BlMsmE | Stachyose | *B. licheniformis* | Binding test  Crystal structure | (Zhang *et al.* 2020) |
|  | Not described | CMMBP | Cyclic α-maltosyl-(1,6)-maltose | *A. globiformis* | Binding test  Crystal structure | (Kohno *et al.* 2020) |
|  | Not described | BpAXBP1 | Xylooligosaccharides (XOS) and AXOS4 (arabinoxylooligosaccharides whose nonreducing end of the xylose backbone was not decorated with arabinose) | *B. pseudocatenulatum* | Transcriptional analysis  Binding test | (Saito *et al.* 2020) |
|  |  | BpAXBP2 | Arabinoxylooligosaccharides (AXOS), including double arabinofuranosyl-decorated XOS (AXOS2) |  |  |  |
|  |  | BpAXBP3 | AXOS2 |  |  |  |
|  | Not described | αGlyBP | Disaccharide α-glycosides | *T. thermophilus HB8* | Crystal structure  Binding test | (Chandravanshi, Gogoi and Kanaujia 2020) |
|  | Not described | βGlyBP | β-glucosides | *T. thermophilus HB8* | Binding test  Crystal structure | (Chandravanshi, Samanta and Prasad Kanaujia 2020) |
|  | Not described | RhLNBBP | Lacto-*N*-biose (LNB), galacto-*N*-biose (GNB), lacto-*N*-tetraose (LNT) | *R. hominis* | Transcriptional analysis  Binding test  Crystal structure | (Pichler *et al.* 2020) |
|  |  | RiLe^a^/^b^BP | Fucosyl-decorated Lewis^b^ (Le^b^) tetraose and Lewis^a^ (Le^a^) triose, lacto-*N*-biose (LNB), galacto-*N*-biose (GNB) | *R. inulinivorans* |  |  |
|  | Not described | GL-BP | Lacto-*N*-biose (LNB), galacto-*N*-biose (GNB) | *B. longum JCM 1217* | Crystal structure | (Fushinobu and Abou Hachem 2021) |
|  |  | FL transporter-2 | Fucosyllactose (FL) | *B. infantis JCM 1222* |  |  |
|  |  | BlAXBP | Xylooligosaccharides (DP2-6), arabinoxylan-oligosaccharides (DP3-5) | *B. animalis* subsp. *lactis* Bl-04 |  |  |
|  |  | BlG16BP | Oligosaccharides sharing a [Gal/Glc]α1,6Glc | *B. except for B. bifidum* |  |  |
|  | Not described | FpMOBP | β-mannooligosaccharides (β-MOS) | *F. prausnitzii SL3/3* | Transcriptional analysis  Binding test | (Lindstad *et al.* 2021) |
|  | Not described | NagB1 and NagB2 | *N*-acetyl-d-glucosamine disaccharides (GlcNAc)_2_, (GlcNAc)_3_ | *Paenibacillus sp. str. FPU-7* | Crystal structure  Binding test | (Itoh *et al.* 2021) |
|  | Not described | AviABCD | Cellulose oligosaccharides (COS) | *C. bescii* | Transcriptional analysis | (Rodionov *et al.* 2021) |
|  |  | MosABC | Mannose oligosaccharides (MOS) |  |  |  |
|  |  | pecXYZ | Pectin oligosaccharides (POS) |  |  |  |
|  |  | hemXYZ | Pectin oligosaccharides (POS) |  |  |  |
|  |  | RhaXYZ | Rhamnogalacturonides |  |  |  |
|  |  | RglFGH | Rhamnogalacturonides |  |  |  |
|  |  | BxgLFG | Glucuronoxylan oligosaccharides (GXOS) |  |  |  |
|  |  | AxoFGE | β-xylooligosaccharide (β-XOS) |  |  |  |
|  |  | XloEFG | β-xylooligosaccharide (β-XOS) |  |  |  |
|  |  | XynUVW | xylooligosaccharide (XOS) |  |  |  |
|  |  | AxuABC | α-xylooligosaccharide (α-XOS) |  |  |  |
|  |  | AxeUVW | Arabinoxylo-oligosaccharides (AXOS) |  |  |  |
|  |  | MalEFG | Maltooligosaccharides (MOS) |  |  |  |
|  |  | KojEFG | α-glucoside disaccharides trehalose and kojibiose |  |  |  |
|  | Not described | inuABCDEF | Inulin (DP of > 8) | *L. delbrueckii JCM 1002^T^* | Transcriptional analysis  TLC  HPLC | (Tsujikawa *et al.* 2021) |
|  | Not described | ESBP (BAD_RS06835), bglE | Copra meal β-manno-oligosaccharides (CM-β-MOS) (DP2, DP3) | *B. adolescentis DSMZ 20083* | Transport assay  Transcriptional analysis  Binding test  Crystal structure | (Mary, Monica and Kapoor 2022) |
|  | Not described | msmEFG | Raffinose, stachyose, melibiose | *Bifidobacterium* | Transcriptional analysis | (Su *et al.* 2022) |
|  | Not described | xuaABC | Arabinoxylan dextrins | *R. cellulolyticum* | Transcriptional analysis  Binding test | (Liu *et al.* 2022) |
|  | Not described | NgcEFG | Chitooligosaccharides, GlcNAc, (GlcNAc)_2_ | *Streptomyces sp. UH6* | Transcriptional analysis | (Duhsaki *et al.* 2022) |
|  |  | DasABC |  |  |  |  |
|  | Not described | CbpB | Cellodextrin (G2 to G5), laminaribiose | *C. thermocellum* | Binding test  Crystal structure | (Yan *et al.* 2022) |
|  | Not described | SBP_MLG_ | Barley β-glucan, mixed-linkage β-glucans (β-1,3/1,4) | *B. producta ATCC 27340* | Targeted gene deletion  ITC  Circular dichroism spectroscopy  AlphaFold prediction | (Singh *et al.* 2023) |
|  | Not described | MusEFGK_2_I | Maltose, maltotriose | *Gardnerella spp.* | Transcriptional analysis | (Bhandari and Hill 2023) |
|  |  | MalXFGK | Maltooligosaccharides and/or maltodextrins | *Gardnerella spp.* |  |  |
|  |  | RafEFGK | Glucosides and galactosides | *G. vaginalis* |  |  |
|  |  | TMSP | Trehalose, maltose, sucrose, and palatinose | *G. vaginalis* |  |  |
|  |  | MalEFG | Maltose and maltodextrins | *G. leopoldii* |  |  |
|  | Not described | GM006568 | Different monosaccharides but also maltose | *S. avermitilis* | Transposon library,  molecular docking, quantification of residual sugars left by engineered strains | (Dong *et al.* 2024) |
|  | Not described | Athe_2310 | Maltose, trehalose | *A. bescii* | Binding tests, molecular modeling | (Tjo *et al.* 2025) |
|  |  | Athe_2574 | Maltodextrins | *A. bescii* |  |  |
| PepT | 3.A.1.5.16 | BglpEFGKL  (TM_0031) | Cellobiose, laminaribiose | *T. maritima* MSB8 | Transcriptional analysis  Binding test | (Conners *et al.* 2005; Nanavati, Nguyen and Noll 2005) |
|  | 3.A.1.5.32 | F0TFS5-9  (LAC30SC_07285) | Stachyose | *L. acidophilus* NCFM | Transcriptional analysis  Gene deletion | (Andersen *et al.* 2012) |

**Supplementary Table 3. List of the bacterial PTS transporters involved in glycoside utilization, characterized either by binding or transport assays, gene deletion and/or transcriptional approaches.**

The glycoside PTS transporters retrieved from the literature but which are not listed in the TCDB were classified based on their conserved domains by using a BLAST analysis against the NCBI’s Conserved Domain Database (Marchler-Bauer *et al.* 2017).

| **TCDB Family name** | **TCID** | **Protein name (locus tag)** | **Substrate** | **Organism** | **Characterization method** | **Literature** |
| --- | --- | --- | --- | --- | --- | --- |
| Glc | 4.A.1.1.3 | MalX  (b1621) | Maltose | *E. coli* K12 | Heterologous expression in *E. coli* strain | (Reidl and Boos 1991) |
|  | 4.A.1.1.8 | MalP  (BSU08200) | Maltose | *B. subtilis* 168 | Transcriptional analysis  Gene deletion | (Yamamoto *et al.* 2001) |
|  | 4.A.1.1.10 | AglA  (FYK33_RS28885) | Isomaltose, trehalulose, maltulose, leucrose, palatinose | *K. pneumoniae* ATCC 23357 | Heterologous express in *E. coli* strain  Transport assay | (Pikis *et al.* 2006) |
|  | 4.A.1.1.11 | MalT  (SMU_2047) | Maltose, maltotriose | *S. mutans* ATCC 700610 | Gene deletion | (Webb, Homer and Hosie 2007) |
|  | 4.A.1.1.12 | MalT (SPy_1986) | Maltose, maltotriose | Group A *Streptococcus* MGAS5005 | Transcriptional analysis  Gene deletion | (Shelburne *et al.* 2008) |
|  | 4.A.1.1.17 | MalT  (BCE33L0344) | Maltose | *B. cereus* E33L | Transport assay  Binding test  Crystal structure | (McCoy *et al.* 2016) |
|  | 4.A.1.2.1 | ScrA | Sucrose | *K. pneumoniae* KAY2026 | Phosphorylation activity | (Titgemeyer *et al.* 1996) |
|  | 4.A.1.2.3 | AscF  (b2715) | Salicin | *E. coli* K-12 | Gene deletion | (Desai, Nandimath and Mahadevan 2010) |
|  | 4.A.1.2.4 | TreB  (b4240) | Trehalose, maltose, sucrose | *E. coli* K-12 | Heterologous expression in other *E. coli* strain  Gene deletion | (Klein, Horlacher and Boos 1995; Steen *et al.* 2019) |
|  | 4.A.1.2.5 | BglF  (cgR_2729) | Methyl-β-glucoside, salicin, arbutin | *C. glutamicum* R | Heterologous expression in *C. glutamicum* | (Kotrba, Inui and Yukawa 2003) |
|  | 4.A.1.2.6 | BglP | Aesculin, arbutin | *S. mutans* NG8 | Gene deletion  Heterologous expression in *E. coli* | (Cote *et al.* 2000) |
|  | 4.A.1.2.9 | SacP  (BSU38050) | Sucrose | *B. subtilis* 168 | Transport assay | (Fouet *et al.* 1987) |
|  | 4.A.1.2.12 | PtsS  (Cgl2642) | Sucrose | *C. glutamicum* ATCC 13032 | Gene deletion | (Moon *et al.* 2005) |
|  | 4.A.1.2.14 | PTS15  (LGAS_1669) | Cellobiose | *L. gasseri* ATCC 33323 | Transcriptional analysis  Gene deletion | (Francl, Thongaram and Miller 2010) |
|  | 4.A.1.2.16 | PTS20  (LGAS_1778) | Sucrose | *L. gasseri* ATCC 33323 | Transcriptional analysis  Gene deletion | (Francl, Thongaram and Miller 2010) |
|  | Not described | scrT  (SP_1722) | Sucrose | *S. pneumoniae* TIGR4 | Gene deletion | (Iyer and Camilli 2007) |
|  | Not described | PTS EIIBCA (lp_0185) | Short-chain fructooligosaccharides (scFOS) | *L. plantarum WCFS1* | Transport assay  Transcriptional analysis | (Saulnier *et al.* 2007) |
|  | Not described | PTS1  (LPST_C0152) | Fructooligosaccharides (DP2-5) | *L. plantarum* ST-III | Transcriptional analysis  Gene deletion | (Chen *et al.* 2015) |
|  | Not described | PTS26  (LPST_C2650) | Fructooligosaccharides (DP2-5) | *L. plantarum* ST-III | Transcriptional analysis  Gene deletion | (Chen *et al.* 2015) |
|  | Not described | PTS EIIBCA | Sucrose | *L. plantarum* | Genomic analysis  Proteomic analysis | (Buntin *et al.* 2017) |
|  |  | FosABCDX PTS | Fructooligosaccharides (FOS) |  |  |  |
|  | Not described | Lac_PTS, LacEF | Lactose | *L. casei, L. paracasei W20* | Transport assay  Transcriptional analysis | (Böger *et al.* 2019) |
|  | Not described | GAG-PTS (EIIABCD) | Unsaturated hyaluronan disaccharide | *S. agalactiae* | Transport assay  Gene deletion  Crystal structure | (Oiki *et al.* 2019) |
|  | Not described | EIIB^Cel^, EIIB^Cel^ | β-1,6-oligosaccharides | *E. faecalis* | Gene deletion  Binding test  Transcriptional analysis | (Grand *et al.* 2019) |
|  | Not described | EIIA/B^Cel1^, EIIA/B^Cel2^ | Cellobiose | *L. monocytogenes* | Gene deletion | (Cao *et al.* 2019) |
|  | Not described | PTS_EIIBCA  (CCG34776) | Kestose | Uncultured *Dorea* | Transcriptional analysis  Gene deletion | (Wang et al., 2020) |
|  | Not described | celBCD | Cellobiose, β-galactosides | *G. stearothermophilus T-1* | Transport assay  Transcriptional analysis | (Shulami *et al.* 2020) |
|  | Not described | bglP | β-glucoside | *S. pyogenes (group A Streptococcus [GAS])* | Gene deletion  Transcriptional analysis | (Braza *et al.* 2020) |
|  | Not described | PTS23C (lp_3010) | Un-substituted galactomannooligosaccharides (GMOS, DP2 and DP3) | *L. plantarum WCFS1* | Transcriptional analysis | (Panwar and Kapoor 2020) |
|  |  | PTS1BCA (lp_0185) | Substituted galactomannooligosaccharides (GMOS, DP2 and DP3) |  |  |  |
|  | Not described | EIIABC^cel2^ | Cellobiose | *T. aotearoense SCUT27* | Transcriptional analysis  Gene deletion | (Qu *et al.* 2021) |
|  | Not described | PTS IIC (scrA) | Fructooligosaccharides | *L. murine (A56)*  *E. hirae (BG)*  *E. faecalis (E1)*  *L. casei (B52)*  *E. gallinarum (B41)*  *S. alactolyticus (B28)* | Transcriptional analysis | (Gu *et al.* 2022) |
|  |  | PTS EIIBCA | Fructooligosaccharides | *L. plantarum (A64)* |  |  |
|  |  | PTS IIBC and IIAB | Fructooligosaccharides | *L. rhamnosus (B63)* |  |  |
|  |  | PTS IIBCA | Fructooligosaccharides | *L. paracasei (B45).* |  |  |
|  | Not described | -  (peg.1635, peg.4961 and peg.1634) | Chitooligosaccharides, GlcNAc | *Streptomyces sp. UH6* | Transcriptional analysis | (Duhsaki *et al.* 2022) |
|  | Not described | PTS IIBCA | Short-chain oligofructose | *L. plantarum QS7T* | Transcriptional analysis | (Zhu *et al.* 2022) |
|  | Not described | ScrA | Sucrose, glucose | *S. thermophilus LMD-9* | Transcriptional analysis  Gene deletion | (Gasser *et al.* 2022) |
|  | Not described | - | Beta-glucoside, cellobiose | *Gilliamella, Lactobacillus Firm-5* | Transcriptional analysis | (Su *et al.* 2022) |
|  | Not described | mini PTS | Cellobiose, glucose, pentoses | *R. cellulolyticum* | Transcriptional analysis  Gene deletion  Binding test  Crystal structure  Phosphorylation activity | (Xu *et al.* 2023) |
|  | Literatur | PTS | Arbutin, cellobiose, fructose, glucose, trehalose | *P. thermoglucosidasius DSM 2542* | Gene deletion | (Bidart, Gharabli and Welner 2023) |
| Lac | 4.A.3.1.2 | PTS 6CB  (LGAS_343) | Lactose | *L. gasseri ATCC*33323 | Transcriptional analysis  Gene deletion | (Francl, Hoeflinger and Miller 2012) |
|  | 4.A.3.1.3 | PTS 9BC (LGAS_501) | Lactose | *L. gasseri* ATCC33323 | Transcriptional analysis  Gene deletion | (Francl, Hoeflinger and Miller 2012) |
|  | 4.A.3.2.1 | ChbC  (b1737) | N,N'-diacetylchitobiose, cellobiose | *E. coli* K-12 | Gene deletion | (Kachroo *et al.* 2007) |
|  | 4.A.3.2.2 | LicC  (BSU38580) | Lichenan oligosaccharides (DP unclear) | *B. subtilis* 168 | Gene deletion | (Tobisch *et al.* 1997) |
|  | 4.A.3.2.5 | ChbC | N,N'-diacetylchitobiose | *S. marcescens* 2170 | Gene deletion | (Uchiyama *et al.* 2003) |
|  | 4.A.3.2.6 | CelB  (VC_1282) | N,N'-diacetylchitobiose | *V. cholerae* serotype O1 ATCC 39315 | Transcriptional analysis  Gene deletion | (Meiborn *et al.* 2004) |
|  | 4.A.3.2.7 | CelC  (KP1_4524) | Cellobiose | *K. pneumoniae* NTUH-K2044 | Gene deletion | (Wu *et al.* 2012) |
|  | 4.A.3.2.8 | CelB  (BCE_5325) | N,N'-diacetylchitobiose | *B. cereus* ATCC 10987 | Transport assay  Gene deletion  Structure | (Cao *et al.* 2011) |
|  | 4.A.3.2.9 | Lactose transporter | Lactose | *K. pneumoniae* CG43 | Transport assay | (Imai and Hall 1981) |
|  | Not described | ChbBCA | Chitin disaccharide (GlcNAc-GlcNAc; N,N′-diacetylchitobiose) and chitin trisaccharide (GlcNAc-GlcNAc-GlcNAc; N,N′,N′′-triacetylchitotriose) | *E. coli K12* | Gene deletion  Transport assay | (Keyhani, Bacia and Roseman 2000; Walter, Friz and Mayer 2021) |
|  | Not described | GenB  (OG1RF_10235) | Gentiobiose, amygdalin | *E. faecalis* OG1RF | Gene deletion Transcriptional analysis | (Grand *et al.* 2019) |
| Man | 4.A.6.1.13 | AlfF  (LCAB_28290) | Fucosyl-α-1,3-*N*-acetylglucosamine | *L. casei* BL23 | Gene deletion | (Rodríguez-Díaz, Rubio-del-Campo and Yebra 2012) |
|  | 4.A.6.1.14 | PTS-IIC  (spr0294) | Hyaluronic acid (DP unclear) | *S. pneumoniae* ATCC BAA-255 | Gene deletion | (Marion *et al.* 2012) |
|  | 4.A.6.1.18 | GnbC  (LCABL_02930) | Galacto *N*-biose and lacto *N*-biose | *L. casei* BL23 | Gene deletion  Transcriptional analysis | (Bidart *et al.* 2014) |
|  | Not described | Mpt PTS | *N*-acetylglucosamine; glucosamine | *E. faecalis* | Gene deletion | (Keffeler *et al.* 2021) |
|  |  | PTS IICBA  (EF1516, NagE) |  |  |  |  |

**Supplementary Table 4. List of the bacterial MFS transporters involved in glycoside utilization, characterized either by binding or transport assays, gene deletion and/or transcriptional approaches.**

The glycoside MFS transporters retrieved from the literature but which are not listed in the TCDB were classified based on the identified conserved domain using a BLAST analysis against the NCBI’s Conserved Domain Database (Marchler-Bauer *et al.* 2017).

| **TCDB Family name** | **TCID** | **Protein name**  **(locus tag)** | **Substrate** | **Organism** | **Characterization method** | **Reference** |
| --- | --- | --- | --- | --- | --- | --- |
| OHS | 2.A.1.5.1 | LacY  (b0343) | Lactose, melibiose,  thio-β-methyl galactopyranoside (TMG), 4-nitrophenyl-β-d-galactopyranoside,  4-nitrophenyl-α-d-galactopyranoside | *E. coli* K-12 | Transport assay  Point mutation  Structure | (King and Wilson 1990; Abramson *et al.* 2003; Kaback 2015) |
|  | 2.A.1.5.2 | RafB  (RAFB_  ECOL) | Raffinose | *E. coli* K-12 | Transport assay  Point mutation | (Camp *et al.* 2007) |
|  | 2.A.1.5.3 | CscB | Sucrose, maltose | *E. coli* EC3132 | Transport assay Heterologous expression in another *E. coli* strain | (Peng *et al.* 2009) |
|  | 2.A.1.5.4 | MelY  (ECL_05077) | Melibiose, lactose | *E. cloacae* ATCC 13047 | Gene deletion  Point mutation  Transport assay | (Shinnick, Perez and Varela 2003; Tavoulari and Frillingos 2008) |
|  | Not described | FosT  (Aec46) | Fructooligosaccharides (DP3-4) | *E. coli* BEN2908 | Gene deletion | (Schouler *et al.* 2009) |
|  | Not described | ydjE | Fructooligosaccharides | *B. longum (SB22)*  *B. pseudolongum (SB12)* | Transcriptional analysis | (Gu *et al.* 2022) |
|  |  | lacY |  | *B. longum (SB22)*  *B. pseudolongum (SB12)*  *S. alactolyticus (B28)* |  |  |
|  | Not described | cscB | Copra meal β-manno-oligosaccharide (CM-β-MOS, DP≥3) | *B. adolescentis DSMZ 20083* | Transport assay  Transcriptional analysis  Binding test  Crystal structure | (Mary, Monica and Kapoor 2022) |
|  | Not described | MalT (AZI09_10325) | Maltose | *L. brevis TMW 1.2112* | Proteomic analysis | (Bockwoldt *et al.* 2022) |
| FHS | 2.A.1.7.6 | ScrT  (Sfri_3989) | Sucrose | *S. frigidimarina* NCIMB 400 | Heterologous expression in *E. coli* | (Rodionov *et al.* 2010) |
| GPH | 2.A.2.1.1 | MelB  (EcE24377A_4674) | Melibiose | *E. coli* E24377A | Transport assay  Binding test  Structure | (Ethayathulla *et al.* 2014) |
|  | 2.A.2.2.1 | LacS  (DID82_  03005) | Lactose | *S. thermophilus* A147 | Heterologous expression in *E. coli*  Transport assay | (Poolman *et al.* 1989) |
|  | 2.A.2.3.1 | GusB  (FAM13_RS00460) | Glucuronide | *E. coli* K-12 | Transcriptional analysis  Transport assay | (Liang *et al.* 2005) |
|  | 2.A.2.3.3 | XylP  (XYLP_  LACP) | Isoprimeverose | *L. pentosus* NZ9000 | Transport assay | (Heuberger, Smits and Poolman 2001) |
|  | 2.A.2.5.1 | TogT | Oligogalacturonides (DP>2) | *E. chrysanthemi* 3937 | Gene deletion | (Hugouvieux-Cotte-Pattat and Reverchon 2001) |
|  | 2. A.2.6.2 | MalY  (CC_2283) | Maltose | *C. crescentus* ATCC 19089 | Gene deletion  Transport assay | (Lohmiller *et al.* 2008) |
|  | Not described | F5_MFS (CCG34981.1) | Xylooligosaccharides (DP2-4) | Uncultured *Bacteroides* | Gene deletion | (Tauzin *et al.* 2016c) |
|  | Not described | LacS  (Lba1463) | Galactooligosaccharides (DP2-6), lactose, lactiol | *L. acidophilus* NCFM | Transcriptional analysis  Gene deletion | (Andersen *et al.* 2011) |
